# Cutting edge rare earth radiometals: prospects for cancer theranostics

**DOI:** 10.1186/s41181-022-00173-0

**Published:** 2022-08-26

**Authors:** Alexander W. E. Sadler, Leena Hogan, Benjamin Fraser, Louis M. Rendina

**Affiliations:** 1 School of Chemistry, The University of Sydney, Sydney, NSW 2006 Australia; 2 ANSTO Life Sciences, Australian Nuclear Science and Technology Organisation (ANSTO), Kirrawee, NSW 2232 Australia

**Keywords:** Rare earth, Lanthanoid, Theranostics, Cancer, Imaging, Therapy, Radionuclide production

## Abstract

**Background:**

With recent advances in novel approaches to cancer therapy and imaging, the application of theranostic techniques in personalised medicine has emerged as a very promising avenue of research inquiry in recent years. Interest has been directed towards the theranostic potential of Rare Earth radiometals due to their closely related chemical properties which allow for their facile and interchangeable incorporation into identical bifunctional chelators or targeting biomolecules for use in a diverse range of cancer imaging and therapeutic applications without additional modification, i.e. a “one-size-fits-all” approach. This review will focus on recent progress and innovations in the area of Rare Earth radionuclides for theranostic applications by providing a detailed snapshot of their current state of production by means of nuclear reactions, subsequent promising theranostic capabilities in the clinic, as well as a discussion of factors that have impacted upon their progress through the theranostic drug development pipeline.

**Main body:**

In light of this interest, a great deal of research has also been focussed towards certain under-utilised Rare Earth radionuclides with diverse and favourable decay characteristics which span the broad spectrum of most cancer imaging and therapeutic applications, with potential nuclides suitable for *α*-therapy (^149^Tb), *β*^−^-therapy (^47^Sc, ^161^Tb, ^166^Ho, ^153^Sm, ^169^Er, ^149^Pm, ^143^Pr, ^170^Tm), Auger electron (AE) therapy (^161^Tb, ^135^La, ^165^Er), positron emission tomography (^43^Sc, ^44^Sc, ^149^Tb, ^152^Tb, ^132^La, ^133^La), and single photon emission computed tomography (^47^Sc, ^155^Tb, ^152^Tb, ^161^Tb, ^166^Ho, ^153^Sm, ^149^Pm, ^170^Tm). For a number of the aforementioned radionuclides, their progression from ‘bench to bedside’ has been hamstrung by lack of availability due to production and purification methods requiring further optimisation.

**Conclusions:**

In order to exploit the potential of these radionuclides, reliable and economical production and purification methods that provide the desired radionuclides in high yield and purity are required. With more reactors around the world being decommissioned in future, solutions to radionuclide production issues will likely be found in a greater focus on linear accelerator and cyclotron infrastructure and production methods, as well as mass separation methods. Recent progress towards the optimisation of these and other radionuclide production and purification methods has increased the feasibility of utilising Rare Earth radiometals in both preclinical and clinical settings, thereby placing them at the forefront of radiometals research for cancer theranostics.

## Introduction

With advances in approaches to cancer therapy and imaging, the application of theranostic techniques for more “personalised” approaches to patient treatment has emerged as a very promising avenue of research inquiry (Marin et al. 2020; DeNardo and DeNardo 2012). The theranostic protocol in nuclear medicine typically involves either the selection and administration of a radionuclide pair of which one is for imaging (e.g. ^68^Ga or ^18^F for PET imaging) and the other for therapy (e.g. ^177^Lu for *β*^−^-therapy) (Filippi et al. 2020), ideally of the same element to minimise pharmacokinetic/biological behaviour differences in vivo (Türler 2019); or the utilisation of a single radionuclide that exhibits favourable decay characteristics that permit dual functionality as both an imaging and therapeutic agent (Türler 2019). As theranostic models of cancer treatment gain momentum both in the research laboratory and in clinical application, the need for a reliable, economical and high purity/yield source of appropriate radionuclides is required. Consequently, it is often the economic/production/availability factors that bolster the popularity of certain radionuclides in the minds of researchers and sustains inquiry into their clinical usefulness, rather than their specific decay characteristics that determine their clinical suitability. However, as clinical demand for certain isotopic decay characteristics increases, the focus shifts to improving the production process and economic utility of certain radionuclides that show significant promise for clinical application.

**Fig. 1:**
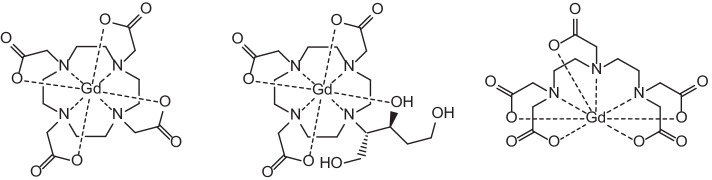
Gadolinium contrast agents (left to right) Gd-DOTA, Gadolinium(III)-tris(carboxymethyl)-1,4,7,10-tetraazacyclododecane-butriol (Gd-BT-DO3A) and Gadolinium(III)-diethylenetriaminepentaacetic acid (Gd-DTPA)

**Fig. 2:**
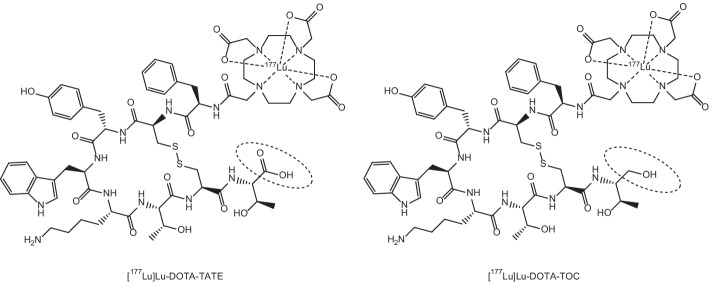
Molecular structures of [^177^Lu]Lu-DOTA-TATE and [^177^Lu]Lu-DOTA-TOC

In recent years, interest has been focussed around the theranostic potential of Rare Earth radionuclides. Of the lanthanoids, they exhibit similar chemical properties due to the filling of the 4*f* electron orbital across the period leaving the 6*s* electrons exposed (Elliott 2013; Cotton 2006). These 4*f* electrons are largely shielded from chemical interaction due to the filled 5*s* and 5*p* orbitals, and this gives rise to similar properties including similar stable oxidation states (generally +3), hard Lewis acid behaviour, affinity for hard donor sites for complexation (O and N) and largely electrostatic bonding (Elliott 2013; Cotton 2006; Moeller et al. 1965; Peacock 2016). These similar chemical properties make them of interest to researchers due to the potential for different lanthanoid radionuclides to be easily and reliably incorporated into the same (or similar) bifunctional chelators or targeting biomolecules for cancer treatment. This significantly simplifies the syntheses of such compounds by employing a “one-size-fits-all” approach through allowing the same ligand scaffold to coordinate different radiolanthanoids in an interchangeable manner depending on the intended imaging or therapeutic purposes (Cotton 2006; Moeller et al. 1965; Cutler et al. 2000). The gold-standard of Rare Earth metal complexation currently is 1,4,7,10-tetraazacyclododecane-1,4,7,10-tetraacetic acid (DOTA) which is easily incorporated into targeting molecule structures and forms very stable complexes with Rare Earth metals as shown by its use in radiotherapeutics such as Lutetium-177-DOTA-Octreotate ([^177^Lu]Lu-DOTA-TATE) or Lutetium-177-DOTA-Octreotide ([^177^Lu]Lu-DOTA-TOC) as well as for chelation of Gd for MRI contrast agents (Cutler et al. 2000; Graf et al. 2020; Staanum et al. 2021; Strosberg et al. 2017; Clough et al. 2019; Yang et al. 2008; Yokoyama and Shiraishi 2020) (see Figs. 1 and 2). Due to the fact that complementary diagnostic and therapeutic radionuclides are frequently incorporated into different targeting molecules/vectors, they can exhibit different biodistribution and pharmacokinetics in the body. Similar chemical characteristics that allow for stable complexation without structural variation to the chelator or targeting molecule have the potential to address (at least in part) issues surrounding differences in biodistribution between imaging radionuclides and therapeutic radionuclides after administration. This is imperative for effective theranostic treatment regimens because the imaging radionuclide and the therapy radionuclide must ideally exhibit near identical biodistribution, selective tumour uptake and bioelimination tendencies in order for tandem theranostic imaging and therapy to work in a complementary manner. Identical bifunctional chelators/targeting biomolecules for complexation of a range of radionuclides (each of which display similar chemical properties in of themselves) significantly reduce the variability in these factors after administration.

In addition to advantages in similar chemical properties, synthesis and clinical administration, certain Rare Earth radionuclides have garnered attention due to their radioactive decay characteristics showing significant potential for theranostic applications (Cutler et al. 2000; Mishiro et al. 2019). These applications tend to fit into three categories: complementary radionuclides for already established imaging or therapy agents; novel radionuclides that have been hamstrung by lack of availability or reliable production facilities/methods to date, or as potential improvements over existing therapies for particularly intractable cases of cancer malignancy.

While aspects regarding developments in radiolanthanoid chelation have been discussed in conjunction with production, radiochemistry and application of popular radiolanthanoids currently progressing towards or already in clinical use (Cutler et al. 2000; Mishiro et al. 2019; Amoroso et al. 2017; Kostelnik and Orvig 2019; Notni and Wester 2018), the state of production and potential application of certain cutting edge radiolanthanoids has not been reviewed in depth. This review with focus on recent progress and innovations in the area of underutilised lanthanoid radionuclides of interest for theranostic applications by providing a snapshot of the state of their production via nuclear reactions, subsequent promising indications of their theranostic capabilities in the clinic, as well as factors that have impacted upon their progress through the development pipeline (see Table 1).

**Table 1:** Decay characteristics and applications for certain Rare Earth radionuclides

Radionuclide	Half-life (h)	Decay characteristics	Average decay particle energy (keV)^a^	Maximum γ energy (keV) (intensity %)^a^	Nuclear reaction(s) for radionuclide production	Applications to nuclear medicine
^43^Sc	3.89	β^+^ (88%) EC (12%)	419	373 (22%)	^40^Ca(α,n)^43^Ti → ^43^Sc ^43^Ca(p,n)^43^Sc ^46^Ti(p,α)^43^Sc	PET
^44^Sc	4.04	β^+^ (94%) EC (6%)	632	1157 (100%)	^45^Sc(p,2n)^44^Ti → ^44^Sc ^44^Ca(p,n)^44^Sc ^44^Ca(d,2n)^44^Sc ^44^Ca(α,3np)^44^Sc	PET
^47^Sc	80.4	β^−^ (100%)	162	159 (68%)	^48^Ca(p,2n)^47^Sc ^44^Ca(α,p)^47^Sc ^46^Ca(n,γ)^47^Ca → ^47^Sc ^47^Ti(n,p)^47^Sc ^48^Ti(γ,p)^47^Sc ^51^ V(γ,α)^47^Sc ^48^Ca(γ,n)^47^Ca → ^47^Sc	β^−^-therapy, SPECT
^149^Tb	4.1	α (17%) β^+^ (7%) EC (76%)	3970 (α) 728 (β^+^)	165 (26%) 352 (29%) 389 (18%) 817 (12%) 853 (16%)	Ta-foil spallation ^152^Gd(p,4n)^149^Tb ^141^Pr(^12^C,4n)^149^Tb ^142^Nd(^12^C,5n)^149^Tb	α-therapy, PET
^152^Tb	17.5	β^+^ (20%) EC (80%)	1142	344 (64%)	Ta-foil spallation ^152^Gd(p,n)^152^Tb	PET
^155^Tb	127.7	EC (100%)		87 (32%) 105 (25%)	Ta-foil spallation ^155^Gd(p,n)^155^Tb ^159^Tb(p,5n)^155^Dy → ^155^Tb	SPECT
^161^Tb	166.8	β^−^ (100%) 12.4 e^−^ per decay	154 (β^−^) 3.75 per AE	26 (23%) 49 (17%) 75 (10%)	^160^Gd(n,γ)^161^Gd → ^161^Tb	β^−^-therapy, Auger electron therapy, SPECT
^132^La	4.6	β^+^ (42%) EC (58%)	1290		^132^Ba(p,n)^132^La ^134^Ba(p,3n)^132^La	PET
^133^La	3.9	β^+^ (7%) EC (93%)	461	278 (2%) 290 (1%) 302 (2%)	^134^Ba(p,2n)^133^La ^135^Ba(p,3n)^133^La	PET
^135^La	18.9	EC (> 99%) 10.6 e^−^ per decay	< 4 per AE	481 (2%)	^135^Ba(p,n)^135^La ^136^Ba(p,2n)^135^La ^137^Ba(p,3n)^135^La	Auger electron therapy
^166^Ho	26.8	β^−^ (100%)	665	80.6 (6%) 1379 (1%)	^165^Ho(n,γ)^166^Ho ^164^Dy(n,γ)^165^Dy(n,γ)^166^Dy → ^166^Ho	β^−^-therapy, SPECT
^153^Sm	46.3	β^−^ (100%)	224	103 (29%)	^152^Sm(n,γ)^153^Sm	β^−^-therapy, SPECT
^165^Er	10.4	EC (100%)	5.3 (65.6%) 38.4 (4.8%)		^166^Er(p,2n)^165^Tm → ^165^Er ^165^Ho(p,n)^165^Er ^165^Ho(d,2n)^165^Er	Auger electron therapy
^169^Er	225.6	β^−^ (100%)	100		^168^Er(*n*,*γ*)^169^Er	β^−^-therapy
^149^Pm	53.0	β^−^ (100%)	363	286 (3%)	^148^Nd(n,γ)^149^Nd → ^149^Pm ^148^Nd(p,γ)^149^Pm ^150^Nd(p,2n)^149^Nd → ^149^Pm ^148^Nd(d,n)^149^Pm ^150^Nd(d,3n)^149^Pm ^150^Nd(γ,n)^149^Nd → ^149^Pm	β^−^-therapy
^143^Pr	326.4	β^−^ (100%)	315		^141^Pr(n,γ)^142^Pr(n,γ)^143^Pr ^142^Ce(n,γ)^143^Ce → ^143^Pr	β^−^-therapy
^170^Tm	3086.4	β^−^ (100%)	317	84 (3%)	^169^Tm(n,γ)^170^Tm	β^−^-therapy, SPECT

^a^Values calculated from the IAEA Live Chart of Nuclides, Nuclear Structure and Nuclear Decay Data; https://www-nds.iaea.org/relnsd/vcharthtml/VChartHTML.html (accessed 16 May 2022)

Notably, Y, Gd and Lu have been discussed at length in other reviews with regards to their production and implementation in theranostic settings, and as such will not be detailed in this review. The reader is encouraged to consult the work of Kostelnik and Orvig (Kostelnik and Orvig 2019), as well as Robertson and Rendina (Robertson and Rendina 2021) for further information on these Rare Earth radiometals.

## ***Scandium: ***^***43***^***Sc, ***^***44***^***Sc, ***^***47***^***Sc***

In recent years, focus on the theranostic concept of chemically identical and matched radionuclide pairs has increased due to the desire to minimise differences in the chemical behaviour between compounds administered to patients, while also simplifying the synthesis of such compounds (Türler 2019; Notni and Wester 2018). Having two radionuclides of the same element incorporated into the same bifunctional chelator molecule (one for diagnostics and one for therapy) ensures that differences in kinetic properties and in vivo biological behaviour are minimal due to identical chemical behaviour (Türler 2019). This allows for the evaluation of therapeutic efficacy using in vivo imaging with the reasonable assumption that biological and chemical behaviour in the body will be more or less identical (Türler 2019; Notni and Wester 2018). Scandium has been proffered as one such element that has suitable radionuclides for diagnostic imaging and therapy, with three theranostically relevant radionuclides that have warranted study for their clinical application: ^43^Sc, ^44^Sc and ^47^Sc (Vaughn et al. 2021). ^43^Sc and ^44^Sc are both suitable for Positron Emission Tomography (PET) imaging due to their respective positron emissions, while ^47^Sc has potential as a *β*^−^-emitter for therapeutic applications that can also be observed using Single Photon Emission Computed Tomography (SPECT) imaging due to its γ-ray emission characteristics (Türler 2019; Snow et al. 2021). PET imaging with ^44^Sc (t_1/2_ = 4.04 h) or ^43^Sc (t_1/2_ = 3.89 h) has been proposed as potentially advantageous over the current use of ^68^Ga due to both radionuclides exhibiting half-lives almost 4 times longer than Ga, which would enable longer acquisition times for PET imaging, better image quality while also enabling the production and transportation of Sc radionuclides over longer distances to more remote facilities requiring radionuclides for PET imaging (Kostelnik and Orvig 2019; Mikolajczak et al. 2021). These radionuclides are also good diagnostic matches for other lanthanoids already in use such as ^177^Lu and ^90^Y due to similarities in their binding and chemical properties (Kostelnik and Orvig 2019). In addition, their chemical nature allows them to be easily chelated by DOTA and DOTA-like analogues and form stable complexes with decreased propensity for demetallation in the body, while also maintaining consistency between the bifunctional chelators/targeting molecule structures used in subsequent therapeutic steps (Kostelnik and Orvig 2019). ^47^Sc has advantageous simultaneous imaging capabilities (SPECT) in conjunction with its *β*^−^-therapy applications, which would allow for its individual use in certain circumstances, but also allows its ideal pairing with diagnostically relevant Sc radionuclides in fulfilment of the chemically identical theranostic concept (Müller et al. 2014a).

Bulwarks to the implementation of Sc radionuclides in matched-pair theranostic applications have arisen in the form of production on the scale and radionuclidic/radiochemical purity required for their seamless translation into the clinic. The production methods of each radionuclide and their limitations are discussed in the context of their theranostic potential.

### ^*44*^*Sc*

^44^Sc has proceeded the most with regards to adequate production and purity, with a number of production methods being utilised with varying degrees of success. Initial forays into its production were by means of a ^44^Ti/^44^Sc generator initially utilising the reliable but indirect ^45^Sc(p,2n)^44^Ti → ^44^Sc nuclear reaction with high proton flux due to long-lived ^44^Ti (Filosofov et al. 2010; Hassan et al. 2018). The first of these generators culminated in the elution of ~ 180 MBq of high purity ^44^Sc in 20 mL of eluate, with a separation factor of 2 × 10^6^ (Filosofov et al. 2010). This method of production also incorporated a post-process washing step to reduce the volume of the eluate and remove contaminants such as oxalate anions (Filosofov et al. 2010; Rösch and Baum 2011; Pruszyński et al. 2010). The ^44^Sc obtained from this process was used to label DOTA-TOC and was subsequently used for the first time in patients (Roesch and Scandium-44, 2012). More recently, generator produced ^44^Sc was used for the first in-human PET imaging of patients with metastasised castration resistant prostate cancer via radiolabelling a prostate-specific membrane antigen (PSMA) targeting ligand (Vipivotide tetraxetan—PSMA-617) (Eppard et al. 2017). Despite the excellent separation of ^44^Sc from the ^44^Ti target, the lack of by-production of ^44m^Sc impurities and the ready use of the resulting radionuclide in clinical studies (Filosofov et al. 2010; Roesch and Scandium-44, 2012; Eppard et al. 2017), comparative studies of indirect vs. direct production methods for ^44^Sc have noted that the post-elution “reverse” purification processes required to limit ^44^Ti breakthrough are challenging and have consequently limited the applicability of this production method outside situations where long ^44^Sc transportation times will be incurred (Hassan et al. 2018). Higher radionuclidic purity is also reported when more direct production methods are used, typically employing cyclotrons (Hassan et al. 2018).

Alternative methods of production have also been achieved using cyclotrons and both natural and enriched Ca targets (Severin et al. 2012; Müller et al. 2013). The natural Ca target route typically yields of up to ~ 650 MBq of ^44^Sc after target irradiation of 1 h, but multiple impurities were noted after the final product was isolated, namely ^44*m*^Sc (the production of which is avoided when utilising the indirect ^44^Ti generator approach), ^47^Sc and ^48^Sc (Severin et al. 2012). The impurities from this production method pose issues for translation into clinical settings due to their long half-lives (^44*m*^Sc: t_1/2_ = 58.6 h, ^47^Sc: t_1/2_ = 80.4 h, ^48^Sc: t_1/2_ = 43.7 h). To avoid such impurities, enriched ^44^Ca targets have typically been used (Müller et al. 2013; Krajewski et al. 2013). These routes use proton, deuteron or *α*-particle irradiation and yield ^44^Sc in adequate yield and radionuclidic purity via ^44^Ca(p,n)^44^Sc, ^44^Ca(d,2n)^44^Sc or ^44^Ca(α,3np)^44^Sc nuclear reactions (Hassan et al. 2018). Respectively, activities of 1900 MBq/50 μA using enriched [^44^Ca]CaCO_3_ powder and 11 MeV protons for 60–90 min (Meulen et al. 2015), and 44 MBq/0.2 μA.h using the same target irradiated with 16.4 MeV deuterons and a 60 min irradiation time (Alliot et al. 2015) have been reported as the most promising for high radionuclidic purity and yield of ^44^Sc, favoured over using other enriched Ca targets such as ^40^Ca, ^42^Ca and ^43^Ca. As a result, production and separation processes (based on extraction chromatography) for such nuclear reactions have been finely tuned to produce high radionuclidic purity ^44^Sc in quantities of up to ~ 2 GBq using optimised proton beams with energies of approximately 11 MeV, and progress in this area of cyclotron-produced ^44^Sc has increased its attractiveness for preclinical and clinical studies due to greater radionuclide availability (Alliot et al. 2015).

### ^*43*^*Sc*

Interest in ^43^Sc as an alternative to ^44^Sc has been invigorated due to the absence of co-emitting high-energy γ-rays compared to ^44^Sc, which pose clinical issues regarding patient radiation dose burdens (Domnanich et al. 2017a; Carzaniga et al. 2017). Similar to the desired production route for ^44^Sc, ^43^Sc has been produced primarily using natural and enriched calcium targets in a cyclotron and irradiation by *α*-particles (Walczak et al. 2015; Szkliniarz et al. 2015, 2016; Minegishi et al. 2016). Typical nuclear reactions employed have been direct: ^40^Ca(α,p)^43^Sc; and indirect: ^40^Ca(α,n)^43^Ti → ^43^Sc. High radionuclidic purities (> 99%) and low impurities (< 1.5 × 10^–5^%) have been consistently reported when using enriched ^40^Ca targets and irradiation periods of 1–2 h (Walczak et al. 2015; Szkliniarz et al. 2015, 2016; Minegishi et al. 2016). Irradiation of natural calcium targets with *α*-particles produced ^43^Sc with activities of ~ 100 MBq/µAh, with the capability of producing radionuclidically pure ^43^Sc at activities on the order of several GBq (Walczak et al. 2015). Optimised separation using UTEVA resin resulted in chemically pure ^43^Sc suitable for PET applications (Walczak et al. 2015). However, the *α*-particle energies required for these production routes range between ~ 24–28 MeV, and the lack of availability of cyclotrons with sufficient high-current *α*-particle beams for such applications has been an impediment to their widespread use (Walczak et al. 2015; Szkliniarz et al. 2016). Another proposed route involves employing enriched ^42^Ca targets and deuteron irradiation using the ^42^Ca(d,n)^43^Sc nuclear reaction, however a similar availability issue is encountered regarding sufficient deuteron beamlines (Walczak et al. 2015; Szkliniarz et al. 2016).

Comparison of production routes using enriched ^43^Ca and ^46^Ti irradiated with protons have also been investigated, using the ^43^Ca(p,n)^43^Sc and ^46^Ti(p,α)^43^Sc nuclear reaction respectively (Domnanich et al. 2017a; Carzaniga et al. 2017). Both routes make use of moderate proton beamline energies ~ 10–15 MeV which are readily available at most commercially operable biomedical cyclotrons (Domnanich et al. 2017a; Carzaniga et al. 2017). Irradiation of enriched ^46^Ti targets has resulted in activity yields of ~ 60–225 MBq after ~ 7 h proton bombardment, levels which are reported to be readily attainable using the above proton beamline energies, and with radionuclidic purity of > 98% ^43^Sc (only 1.5% ^44^Sc and < 0.08% all other Sc isotopes) (Domnanich et al. 2017a). On the other hand, the enriched ^43^Ca target route produced the desired ^43^Sc at higher activity yields than the ^46^Ti target, but with the trade-off that radionuclidic purity was lower and dependent on the enrichment level of the target material (Carzaniga et al. 2017). Using 58% enriched ^43^Ca targets, irradiation caused the simultaneous production of both ^43^Sc and ^44^Sc, resulting in a ratio of ~ 67% ^43^Sc and ~ 33% ^44^Sc with a ^43^Sc activity yield of ~ 250–480 MBq/µAh, while another investigation reported final activity yields of ~ 130 MBq, with near identical ^43^Sc/^44^Sc ratios in the final product, and noted that using lower impinging proton beam energies allowed for an increase the final purity at the cost of overall yield (Carzaniga et al. 2017). Higher yields reported when using enriched calcium targets have been partially attributed to the higher nuclear reaction cross-section of ^43^Ca compared to ^46^Ti (~ 250–275 mb and ~ 40 mb respectively) (Domnanich et al. 2017a; Carzaniga et al. 2017). The weighing up of the relevant advantages and drawbacks of each of these production routes has shown that the ^43^Ca route has greater applicability for routine production of larger yields of ^43^Sc for radiopharmaceutical applications, since target material recycling and comparatively less complicated target preparation and separation procedures are able to mitigate the cost of enriched ^43^Ca (Domnanich et al. 2017a; Carzaniga et al. 2017).

### ^*47*^*Sc*

A number of nuclear reactions using a variety of facilities have been investigated for the production of ^47^Sc. Cyclotrons, nuclear reactors and linear accelerators have been used in this regard, utilising protons, neutrons and *α*-particles for target material bombardment (Minegishi et al. 2016; Misiak et al. 2017; Domnanich et al. 2017b; Kolsky et al. 1998; Bartoś et al. 2012). Cyclotron production of ^47^Sc has been studied via the ^48^Ca(p,2n)^47^Sc nuclear reaction with an optimum proton energy range of 24–17 MeV and an irradiation time of 5 h using a natural CaCO_3_ target (Misiak et al. 2017). Separation of ^47^Sc from the target was achieved using Chelex-100 and UTEVA resins as well as filtration using a 0.2 µm filter. Optimum recovery of Sc isotopes (96%) was achieved using a 0.2 µm filter in 15 min, whereas the extraction resins exhibited lesser recoveries (85% and 79% Sc recovery respectively) over 30 min (Misiak et al. 2017). Activity yields reported using the natural Ca target were ~ 144 kBq/µAh at the optimum proton beam energy, but with only 87% radionuclidic purity (Misiak et al. 2017). These results are partially attributable to the low percentage of ^48^Ca present in natural Ca (0.187% natural abundance). Enriched ^48^Ca targets have been entertained as a solution to the low activity yields reported, but the prohibitive cost of ^48^Ca enrichment has stalled the study of this route, despite the potential to produce ^47^Sc at activity levels on the order of GBq (Misiak et al. 2017). An alternative method of production using a cyclotron involves an enriched ^44^CaO target (97% ^44^Ca) and *α*-particle irradiation via the ^44^Ca(α,p)^47^Sc nuclear reaction and using a precipitation method with a 0.22 µm sterile filter (1.5 h separation) (Minegishi et al. 2016). Even with a highly enriched target material, the activity yield 1.5 h after the end of bombardment (EOB) was ~ 780 kBq/µAh and radionuclidic purity post-separation was lower than that reported when using a ^48^Ca target (22.9% ^47^Sc vs. 73.5% ^43^Sc at EOB, but rising gradually to a maximum 85% at ~ 35.6 h after EOB as dominant ^43^Sc decayed) (Minegishi et al. 2016). These were attributed to the relatively long half-life of ^47^Sc and low production yields (Minegishi et al. 2016; Misiak et al. 2017).

High thermal neutron flux reactor production routes have been used in conjunction with enriched ^46^Ca targets using the indirect ^46^Ca(n,γ)^47^Ca → ^47^Sc nuclear reaction to produce ^47^Sc using a “pseudo-generator-like” approach similar to that employed in the production of ^44^Sc (Müller et al. 2014a; Domnanich et al. 2017b). This approach has shown promise, with one study reporting up to 2 GBq of ^47^Sc being feasibly isolated, while optimisation of the post-production separation and isolation procedures, along with repeated separation of in-grown ^47^Sc from the target material over the following days, allowed for 1.5 GBq to be isolated with a radionuclidic purity exceeding 99.99% in a ~ 700 µL fraction (Domnanich et al. 2017b). This investigation reported the first reliable and reproducible production of ^47^Sc of sufficiently high yield and radionuclidic purity for clinical applications by way of thermal neutron bombardment of enriched ^46^Ca targets. This development serves to potentially mitigate the high cost of the enriched ^46^Ca target material through the implementation of efficient separation and target material recovery methods, and highlights the potential for ^47^Sc to be produced in the necessary quantities and with the necessary purity for radiopharmaceutical purposes in the future (Domnanich et al. 2017b). Comparison of this enriched ^46^Ca method with fast neutron bombardment of an enriched ^47^Ti target was also reported in the same study (Domnanich et al. 2017b). Lower radionuclidic purities were reported using the ^47^Ti(n,p)^47^Sc nuclear reaction, along with significant long-lived ^46^Sc impurities ranging from 3.8 to 11.5% which were dependent on the irradiation period and neutron energy (Domnanich et al. 2017b; Bokhari et al. 2010; Zerkin and Pritychenko 2018). These detracting factors were deemed unfeasible for the production of ^47^Sc with sufficient purity and yield for radiopharmaceutical purposes, hereby bolstering the attractiveness of the enriched ^46^Ca route (Müller et al. 2014a; Domnanich et al. 2017b). Other studies have supported these conclusions (Kolsky et al. 1998; Bartoś et al. 2012). A similar investigation using an enriched ^48^Ti target and proton irradiation via the ^48^Ti(p,2p)^47^Sc nuclear reaction encountered analogous issues of ^46^Sc impurities (Srivastava 2012).

Alternative inquiries into ^47^Sc have been conducted by employing linear accelerators using both natural and enriched Ti as well as Ca targets with γ-ray irradiation. Investigation of enriched ^48^Ti targets using the ^48^Ti(γ,p)^47^Sc nuclear reaction has been reported, with comparison to ^51^ V(γ,α)^47^Sc (Yagi and Kondo 1977). The former was initially deemed superior to the latter despite the near 100% natural abundance of ^51^V due to unavoidable co-production of ^46^Sc and ^48^Sc, as well as low yields of the desired radionuclide when using the ^51^V target (Yagi and Kondo 1977).

More recently, due to the issue of high prices for enriched Ca and Ti starting materials, the ^51^ V(γ,α)^47^Sc reaction has been revisited using high-energy photons from electron linear accelerators has allowed for the production of carrier-free ^47^Sc at purity levels of > 99.998% and 98.8% using 20 MeV and 38 MeV bremsstrahlung energies, respectively (Snow et al. 2021). With appropriate method development, it has been theorised that the required therapeutic dose quantities of 3700 MBq of ^47^Sc could be feasibly produced using this method, with the 20 MeV energy requirements being attainable at nuclear medicine facilities for potential “dose-on-demand” (Snow et al. 2021).

However, the ^48^Ti(γ,p)^47^Sc nuclear reaction is still marred by the same impurities of ^46^Sc and ^48^Sc at percentages of 1.3 and 10.3% of the ^47^Sc activity at the end of irradiation (EOI), with a number of other Sc isotopes also present (Yagi and Kondo 1977). Purity and yields with low to negligible ^46^Sc and ^48^Sc contaminants could only be improved with irradiation energies above 40 MeV with highly enriched [^48^Ti]TiO_2_ targets. Irradiation of said target with 30, 40, 45 and 60 MeV bremsstrahlung energies showed best results were achieved at 60 MeV, with ^46^Sc and ^48^Sc impurities at 0.57 and 0.26% respectively 12 h post-EOI (Yagi and Kondo 1977). It has been reported elsewhere more recently that maximising ^47^Sc production using this reaction is possible with 22 MeV irradiation energies, while also minimising the co-production of other Sc isotope impurities (Mamtimin et al. 2015). Also more recently, natural TiO_2_ targets irradiated with Bremsstrahlung photons were able to yield ^47^Sc at 4.25 MBq/g.kW.hr and 6.92 MBq/g.kW.hr using 35 and 40 MeV beam energies, with the prospect of producing up to ~ 2.96 GBq and ~ 4.81 GBq of ^47^Sc using the same beam energies with a 5 g target (Rotsch et al. 2018).

The indirect ^48^Ca(γ,n)^47^Ca → ^47^Sc nuclear reaction has also been investigated using electron linear accelerators, with results showing the plausibility of high specific activity ^47^Sc being able to be produced via this method (Rane et al. 2015; Starovoitova et al. 2015). The optimum electron beam energy for ^47^Ca production was calculated as 40 MeV in one study, with the size of the target reported as playing an integral role in the resulting specific activity of ^47^Sc produced (Rane et al. 2015). Smaller target sizes were said to result in higher average photon flux through the target, hereby increasing the specific activity produced (Rane et al. 2015). However, it was highlighted in another study that accurate and reliable photonuclear cross sections are necessary for ensuring optimum electron beam parameters as well as correctly predicting yields of desired radionuclides and minimising the co-production of radionuclide contaminants (Starovoitova et al. 2015).

## ***Terbium: ***^***149***^***Tb, ***^***152***^***Tb, ***^***155***^***Tb and ***^***161***^***Tb***

Terbium has been dubbed the 'Swiss army knife' of nuclear medicine due to the wide range of radioisotopic tools possible from this element. The medically relevant radionuclides ^149^Tb, ^152^Tb, ^155^Tb and ^161^Tb cover all the bases: alpha, beta, and Auger emissions suitable for therapeutic applications (^149^Tb, ^161^Tb), and positron and gamma emissions for diagnosis (^152^Tb, ^155^Tb) making terbium an ideal candidate for theranostic applications.

### ^*149*^*Tb*

Alpha radionuclides have garnered significant interest in nuclear medicine due to their ability to deliver a high radiation dose to tumours. This property has allowed for the treatment of a variety of diseases including neuroendocrine tumours (Navalkissoor and Grossman 2019) and, most successfully, prostate cancer with bone metastases (Skelton et al. 2020). The alpha emitting radionuclides most used in these treatments (^225^Ac, ^223^Ra) have alpha emitting daughter products. ^149^Tb is unique in that it has only has a single alpha emission in its decay pathway decreasing the potential impacts of off-target radiation dose to patients. Due to this property, it has attracted research interest for its potential application in targeted *α* settings, notably in folate receptor targeted *α*-therapy (Müller et al. 2012, 2014b), as well as certain radioimmunotherapy (RIT) applications (Beyer et al. 2004a). Other notable properties of ^149^Tb include a relatively short half-life (T_1/2_ = 4.1 h), low *α*-energy (3.97 MeV, I_*α*_ = 16.7%), and a positron emission (E_β+,mean_ = 730 keV, I_β+_  = 7.1%) that may allow, via the use of quatitative PET imaging, the acquisition of patient dose distribution data during therapeautic administration of the radionuclide (Müller et al. 2016). ^149^Tb can be produced via three main pathways (1) proton induced spallation, (2) heavyion induced nuclear reactions (e.g. ^12^C), and (3) light ion induced (e.g. proton or ^3^He) nuclear reactions (Beyer et al. 2002).

**Fig. 3:**
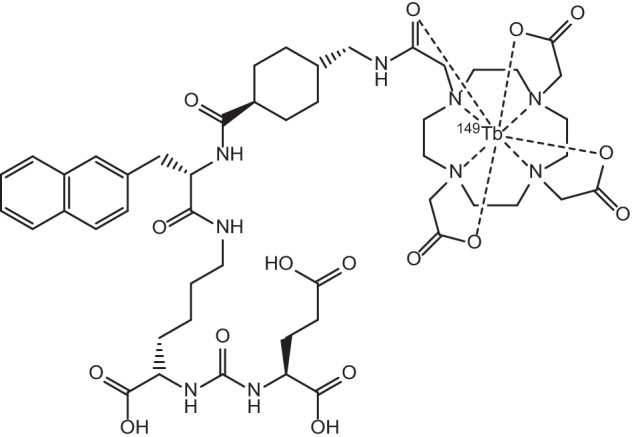
Molecular structure of [^149^Tb]Tb-PSMA-617

The proton induced spallation of Ta targets has been used for many decades to produce a range of radionuclides. Relatively large amounts of ^149^Tb are able to be produced by the nuclear reaction Ta(p,x)^149^Tb, however on-line mass separation processes are required to yield a product of high radionuclidic purity. This method utilises thick Ta targets to compensate for the overall lower neutron capture cross sections that are characteristic for radiolanthanoid production using this technique, and has been calculated to potentially produce up to 19 GBq/μA with a 100 g/cm^2^ target and proton beam energies on the order of 100 μA (Beyer et al. 2002). ^149^Tb produced via this method has recently been used in the preclinical analysis of [^149^Tb]Tb-DOTA-1-NaI3-octreotide ([^149^Tb]Tb-DOTA-NOC) and [^149^Tb]Tb-PSMA-617 (see Fig. 3), as suitable [^149^Tb]TbCl_3_ formulations were readily obtained for radiolabelling after on-line mass separation (Müller et al. 2016; Umbricht et al. 2019). Radiochemical purity (> 98%) and specific activity (5 MBq/nmol) of [^149^Tb]Tb-DOTA-NOC have been achieved, while [^149^Tb]Tb-PSMA-617 was prepared at > 98% radiochemical purity at 6 MBq/nmol levels, both formulations of which were suitable for preclinical studies on AR42J tumour-bearing mice (Müller et al. 2016) and PSMA-positive PC-3 PIP tumour-bearing mice (Umbricht et al. 2019), respectively.

Of the lightion induced production methods, the bombardment of ^151^Eu with 70 MeV ^3^He can yield 150 MBq/μA of ^149^Tb during an 8 h irradiation which is appropriate for therapeutic applications (Moiseeva et al. 2020). The radiochemical purification of ^149^Tb from the Eu target is relatively straightforward however the limited number of high-intensity ^3^He beams worldwide significantly limits potential production via this method. Alternatively, proton bombardment of ^152^Gd can yield 2600 MBq/uAh ^149^Tb via the ^152^Gd(p,4n)^149^Tb nuclear reaction, however the lack of availability of sufficiently enriched ^152^Gd target material (only 0.2% natural abundance) has restricted the feasibility of this approach, as more refined target enrichment is necessary for worthwhile production of ^149^Tb via this method (Beyer et al. 2002).

Heavy ion induced ^149^Tb production can also be achieved via direct ^141^Pr(^12^C,4n)^149^Tb and indirect ^142^Nd(^12^C,5n)^149^Dy → ^149^Tb routes. Quantities of ^149^Tb suitable for antibody radiolabelling have been produced using these methods, after the removal of impurities and bulk target material via cation-exchange chromatography on an Aminex A5 column (60 mm, 3 μm, ~ 13 μm particle size) with α-hydroxyisobutyric acid (α-HIBA) as eluent and subsequent reconstitution in HCl for the final formulation of [^149^Tb]TbCl_3_ (Sarkar et al. 1999).

### ^*155*^*Tb*

^155^Tb undergoes radioactive decay exclusively by electron capture (EC) and has two gamma emissions at 87 keV (32%) and 105 keV (25%) that are suitable for SPECT imaging. The longer half-life (5.32 d) makes it particularly suitable for delivery vectors with longer biological half-lives such as antibodies and large proteins. The ^155^Tb radionuclide as a SPECT agent has theranostic application as a companion dosimetry and treatment planning tool for ^149/161^Tb labelled radiotherapeutics. Preclinical evaluation of [^155^Tb]Tb-DOTA-TOC in mice bearing neuroendocrine tumours has been undertaken (Müller et al. 2014c). ^155^Tb has been produced by numerous methods including *α*-irradiation of Eu targets (Levin et al. 1977), via photonuclear reactions using an EA-25 electron accelerator (Levin et al. 1981), and by proton and deuterium bombardment of Gd targets on 11 to 22 MeV cyclotrons (Dmitriev et al. 1989; Favaretto et al. 2021). Notably, ^155^Tb has been produced at the CERN-MEDICIS facility via spallation of a high purity Ta-foil target with 1.4 GeV protons, after which online mass separation of *m/z* 155 was used to collect approximately 20 MBq of the desired ^155^Tb (along with other isobars) onto a Zn-plated Au foil. Ion-exchange and extraction chromatography were subsequently used to isolate ^155^Tb from isobaric impurities, which gave a final ^155^Tb formulation with radionuclidic purity > 99.9% that was deemed suitable for pre-clinical use (Webster et al. 2019).

### ^*152*^*Tb and matched pairs with*^*149/155/161*^*Tb*

^152^Tb is a positron emitting radionuclide with a relatively long half-life (T_1/2_ = 17.5 h) and relatively high-energy positron emissions (E_β+_mean = 2795 keV, 13.9%) and has potential as a SPECT radionuclide due to multiple gamma emissions. The high positron emission energy, however, leads to lower spatial resolution compared to established PET radionuclides such as ^18^F and ^11^C. These high-energy positron emissions in combination with an array of gamma emissions raises some concerns for radiation burden for patients and workers. ^152^Tb is used as a tool for dosimetry and treatment monitoring for ^149/161^Tb radiotherapeutics and being a PET radiolanthanoid it also has potential application as a companion PET diagnostic/treatment planning tool for ^153^Sm and ^165^Dy radiotherapeutics. ^152^Tb has been used in combination with ^149/155/161^Tb labelled DOTA-conjugates targeting the folate receptor (Chopra 2004), in radioimmunoconjugates for targeted *α*-therapy for malignant melanoma (Rizvi et al. 2000), and in the first-in-human application for radiotherapy of neuroendocrine tumours as [^152^Tb]Tb-DOTA-TOC (Baum et al. 2017). ^152^Tb is produced by heavy ion reactions and proton-induced spallation of Ta targets followed by isotopic separation (Allen et al. 2001), but the production of ^152^Tb in quantities useful for clinical applications remains a significant challenge for light charged particle and heavy ion (HI) activation (Naskar and Lahiri 2021).

## ***Lanthanum: ***^***132***^***La, ***^***133***^***La and ***^***135***^***La***

**Fig. 4:**
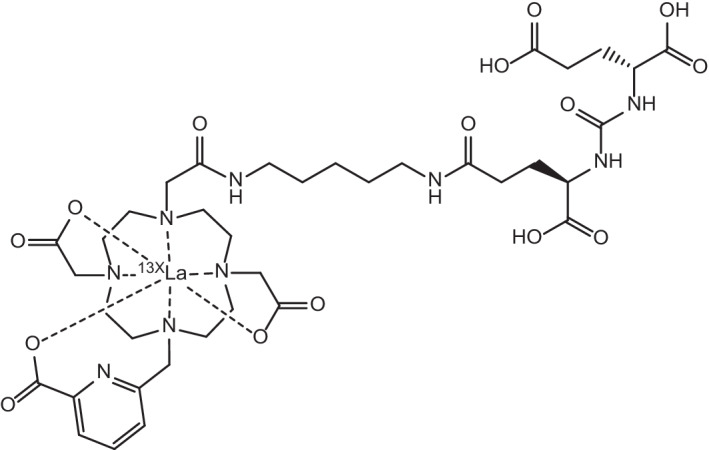
Molecular structure of [^13X^La]La-DO3Apic-DUPA, where X = 2, 5

**Fig. 5:**
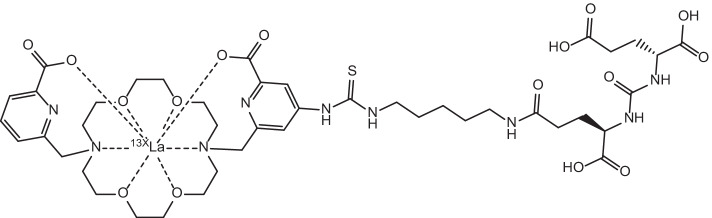
Molecular structure of [^13X^La]La-macropa-DUPA, where X = 2, 5

The trio of lanthanum radionuclides ^132^La, ^133^La and ^135^La present an interesting prospect for the fulfilment of the ideal theranostic concept of chemically-identical matching of diagnostic and therapeutic radionuclides (Aluicio-Sarduy et al. 2020). ^132^La (t_1/2_ = 4.6 h) is a positron emitter that has garnered interest as a PET imaging radionuclide and has seen applications as a surrogate imaging agent to probe the in vivo biodistribution of ^225^Ac due to similarities in their chemical properties (Aluicio-Sarduy et al. 2021). Likewise, the positron emitter ^133^La has also emerged as a PET imaging candidate with the potential to improve the PET imaging quality of metastases and smaller tumours. This is due to the lower maximum positron emission energies exhibited by ^133^La, which result in greater spatial resolution in PET imaging compared to using other radionuclides of interest such as ^68^ Ga and ^44^Sc (Nelson et al. 2020). Its longer half-life (t_1/2_ = 3.9 h) than the commonly-used ^18^F and ^11^C PET agents also presents ^133^La as a longer-lived alternative for the PET imaging/monitoring of longer biological processes (Nelson et al. 2020). Their therapeutic counterpart, ^135^La (t_1/2_ = 18.9 h), has also attracted research interest due to its Auger electron emissions with high linear energy transfer (LET) that provide an attractive alternative to traditional *β*^−^-therapy due to their shorter tissue range and higher number of ionisation events per unit distance travelled being ideally suited for a more targeted approach (Aluicio-Sarduy et al. 2020). Their reduced tissue range and increased propensity for double strand breaks in DNA also makes them ideal for the treatment of smaller tumours and metastases, as they cause more localised damage and reduce collateral damage to healthy tissue (Aluicio-Sarduy et al. 2020; Fonslet et al. 2017). Their potential as theranostic pairs has been investigated through their complexation by 10-((6-carboxypyridin-2-yl)methyl)-1,4,7,10-tetra-azacyclododecane-1,4,7-triacetic acid (DO3Apic) and *N*,*N*’-bis[(6-carboxy-2-pyridil)methyl]-4,13-diaza-18-crown-6 (macropa) chelators and incorporation into PMSA-targeting agent 2-[3-(1,3-dicarboxypropyl)ureido]pentanedioic acid (DUPA) (see Figs. 4 and 5) for the in vivo imaging of PSMA-expressing xenografts in mice (Aluicio-Sarduy et al. 2020).

Production of La radionuclides has been achieved using natural Ba targets with cyclotrons (Fonslet et al. 2017). The production of ^135^La via the proton irradiation of natural Ba targets was achieved using 16.5 MeV proton beam energies and irradiation times of 235–280 min (Fonslet et al. 2017). This production method resulted in the formation of a number of La isotopes, however the short-lived nature of ^134^La (t_1/2_ = 6.5 min), ^136^La (t_1/2_ = 9.9 min), and slightly longer-lived ^132^La (t_1/2_ = 4.5 h) and ^133^La (t_1/2_ = 3.9 h) allowed for the targets to be left to decay for 12–24 h to increase the overall activity of ^135^La and minimise contamination with other isotopes. Purification consisted of dissolution in aqueous HCl, heating, pH adjustment and CM cation-exchange resin with 0.1 M HCl for separation and elution of purified ^135^La (Fonslet et al. 2017). Average production amounts had activity yields of ~ 407 MBq/µAh with radionuclidic purities of 98% at 20 h after irradiation. This method employed chemical separations capable of recovering > 98% of ^135^La produced with an effective molar activity of ~ 70 GBq/µmol in its final formulation (Fonslet et al. 2017). As such, the high activity, radionuclidic purity and yield of the ^135^La isolated were sufficient for the intended clinical applications, and the use of medical cyclotrons already in operation also makes this production route favourable for the production of clinically relevant amounts of ^135^La in the future (Fonslet et al. 2017). However, this study noted a relatively modest separation factor of ~ 10^2^, but that the high labelling effective specific activity obtained rendered the scrupulous separation of ^135^La unnecessary (Fonslet et al. 2017). It was also noted that enriched ^135^Ba targets would increase overall production yield and radionuclidic purity.

The use of enriched Ba targets in the form of [^135^Ba]BaCO_3_ (94.9% ^135^Ba enrichment) in conjunction with cyclotrons has also been investigated for the production of no-carrier-added (n.c.a) ^135^La (Mansel and Franke 2015). This study reported radiochemical yields of ~ 83% and activity yields of ~ 43 MBq using 18 MeV beamline energies and separation using La selective resins, however these results were not suitable for clinical applications and are not of the commercially viable scale necessary for such applications (Mansel and Franke 2015). As reported elsewhere, for production routes involving enriched ^135^Ba to become more viable, significant target development is required to permit the irradiation of an enriched barium oxide or salt, as well as improvements to allow a reduction in ^135^Ba^2+^ recycling after the separation process (Fonslet et al. 2017). In addition, separation procedures for the radiochemical isolation of La radionuclides have been few, and those employed so far have resulted in moderate chemical purity and separation factors (Fonslet et al. 2017; Mansel and Franke 2015; Aluicio-Sarduy et al. 2019). This has resulted in focus on the production of ^135^La as a matched radionuclide pair with both ^132^La and ^133^La from natural Ba targets for a more seamless incorporation of the theranostic concept into current bifunctional chelators and targeting vectors for further applications.

Cyclotrons have also been used for the production of ^132^La from natural Ba targets via the ^132^Ba(p,n)^132^La chemical reaction, in a similar manner to the production of ^135^La, for the production of the theranostically relevant ^132^La/^135^La radionuclide pair (Aluicio-Sarduy et al. 2021, 2019). However, it has been reported that current production routes to ^132^La using natural Ba targets have been hindered by current cyclotron production methods requiring long irradiation times and the relatively low natural abundance of the requisite ^132^Ba isotope (0.1% natural abundance) (Nelson et al. 2020). These production route issues, in conjunction with potentially unfavourable decay characteristics such as the high-energy positron emission of ^132^La resulting in reduced spatial resolution for PET imaging (particularly small tumours), and the high abundance of γ-ray emissions which could result in dose tolerance issues in patients and transportation/handling problems, have led to the investigation of ^133^La as a less production-intensive and more easily handled/administered alternative (Nelson et al. 2020). The ^133^La/^135^La theranostic pair were efficiently produced in a more recent work via natural Ba targets with proton bombardment in medical cyclotrons, in an analogous way to the previously reported production of the ^132^La/^135^La pair (Nelson et al. 2020; Aluicio-Sarduy et al. 2019). This study made use of a new type of natural Ba metal target which was completely encapsulated in an Ag disc covered with Al foil, with assembly being simple and components reusable (Nelson et al. 2020). Targets were irradiated with 22 MeV proton beams for 25–200 min before separation of the desired radionuclide pair using a modified method similar to that used previously for the separation of the ^132^La/^135^La radionuclide pair (Nelson et al. 2020). Formation of ^131^La and ^132^La were observed in small amounts, and ^134^La and ^136^La were observed in significant amounts at EOB, but soon decayed owing to their short half-lives (6.45 and 9.87 min respectively) (Nelson et al. 2020). The resulting radionuclide pair was recovered consistently at > 88% after the automated separation (~ 35 min), and the activity yields were ~ 231 MBq ^133^La and ~ 166 MBq ^135^La at EOB, with saturated yields of ~ 161 MBq/µA ^133^La and ~ 561 MBq/µA ^135^La. These values were an order of magnitude higher than those reported for the ^132^La/^135^La pair (Nelson et al. 2020; Aluicio-Sarduy et al. 2019). Radionuclidic purity and yield were high, and the ^132^Ba(p,n)^132^La nuclear reaction was largely avoided due to low isotopic abundance of ^132^Ba in the target material and a low reaction cross-section over the energy range used. The radionuclide pair was produced with sufficient activity yield and radionuclidic purity for radiolabelling applications with DOTA and macropa resulting high incorporation reported for both chelators (Nelson et al. 2020). This method enabled the production and isolation of clinically relevant activities of ^133^La/^135^La using low cyclotron beam energies with shorter irradiation times than those needed for ^132^La/^135^La, and without the added expense of isotopically enriched Ba (Nelson et al. 2020). It was also noted that enrichment of the Ba target to remove the ^132^Ba isotope would improve the purity even more due to the effective removal of the ^132^La impurity arising from the ^132^Ba(p,n)^132^La reaction. This would also remove any ^131^La arising from the decay of ^132^La, leaving only the desired radionuclide pair after a decay period of 3 h. Adopting such targets would also lower the cyclotron energy required for the production of radionuclidically pure ^133^La/^135^La (Nelson et al. 2020).

## ***Holmium: ***^***166***^***Ho***

^166^Ho has seen a wide range of applications in cancer therapy over the last few decades (Shi et al. 2017). A wide array of therapeutic applications have been investigated with pre-clinical and clinical trials and studies being reported for radiopharmaceutical therapies for bone marrow cancer (Bayouth et al. 1995a, b; Giralt et al. 2003; Christoforidou et al. 2017), metastatic bone pain palliation (Voorde et al. 2019; Bahrami-Samani et al. 2010), brain cancer (Huh et al. 2005; Ha et al. 2013), liver cancer (Cho et al. 2005; Kim et al. 2006), and prostate cancer (Seong et al. 2005; Kwak et al. 2005), among others. This radionuclide has gained attention for its potential theranostic applications due to its favourable decay characteristics that allow for its use as both a therapeutic agent and an imaging agent (Tan et al. 2020). ^166^Ho has a physical half-life of 26.8 h and emits *β*^−^-particles [E_βmax_ = 1.854 MeV (50.0%) and 1.774 MeV (48.7%)] suitable for *β*^−^-therapy (Voorde et al. 2019), while also producing γ-emissions (80.6 keV, 6.2%) that can be used for γ-scintigraphy or SPECT without an excessive dose burden to the patient or radiation damage during transportation, handling, storage or administration since the γ-emissions are not high-energy. In addition, the highly paramagnetic nature of ^166^Ho (4*f*^11^ with 3 unpaired electrons) has led to its use as a magnetic resonance imaging (MRI) contrast agent (Shi et al. 2017; Tan et al. 2020; Vente et al. 2007). These properties allow for the unification of both aspects of the theranostic concept in one radionuclide, which has advanced ^166^Ho as a promising option over the more readily-researched theranostically-matched radionuclide pair approach, due to the alleviation of the need for investigation into theranostic counterparts that satisfy the requirements of similar chemical properties, production availability, chelation kinetics, biodistribution, pharmacokinetics and toxicity concerns, among others. Furthermore, ^166^Ho has the additional apparent advantage of a comparably less complicated nuclear reaction target enrichment process, due to the availability of the requisite parent isotope, ^165^Ho, in 100% natural abundance (Shi et al. 2017; Bahrami-Samani et al. 2010). This has led to ^166^Ho production using the ^165^Ho(n,γ)^166^Ho nuclear reaction, and has allowed for (in combination with a thermal neutron capture cross-section of 64.7 b) relatively high specific activity yields of ^166^Ho (2–5 GBq/mg) to be produced (Mishiro et al. 2019; Iaea-Tecdoc 2003; Zolghadri et al. 2013; Yousefnia et al. 2014). This method typically utilises neutron beam energies ~ 4 × 10^13^ n/cm^2^/s and irradiation times of from 20 to 60 h (Iaea-Tecdoc 2003; Zolghadri et al. 2013; Yousefnia et al. 2014). It has however been reported elsewhere that issues arise with this method due to the resulting ^166^Ho being carrier-added and hence not suitable for certain radiolabelling applications, as well as the long-lived ^166*m*^Ho impurity (t_1/2_ = 1200 y) (Voorde et al. 2019). Moreover, despite the thermal neutron cross-section of this nuclear reaction resulting in high activity levels of the desired radionuclide, specific activity levels are lower than desired at saturation yields due to only a small proportion of the target material undergoing neutron capture and converting to ^166^Ho (Voorde et al. 2019). As a result, the ^166^Ho produced at these modest specific activities cannot be used for the purposes of radiolabelling target molecules (Voorde et al. 2019).

Moreover, radiopharmaceuticals based on ^166^Ho have often encountered issues with dissemination to the relevant medical facilities that require them due to the relatively short half-life of ^166^Ho (t_1/2_ = 26.8 h) (Voorde et al. 2019). The production sites for ^166^Ho, namely nuclear reactors, are typically located further away from most medical facilities, and consequently the ^166^Ho is only able to be transported to facilities within a small radius of the production site due to its disadvantageous half-life (Voorde et al. 2019). This method of production resulting in carrier-added ^166^Ho with lower-than-required specific activities encounters the issues of requiring direct access to a nuclear reactor for radionuclide production and transportation. In addition, the long irradiation times required by this method (typically ~ 60 h) are not ideal. This has led to other production methods being investigated, namely a ^166^Ho radionuclide ‘generator’ approach using ^164^Dy as target material for no-carrier-added ^166^Ho (Vosoughi et al. 2017a, b).

Alternative methods of this nature use ^164^Dy as the target material in conjunction with a double neutron capture reaction ^164^Dy(n,γ)^165^Dy(n,γ)^166^Dy → ^166^Ho (Dadachova et al. 1994; Lahiri et al. 2004). This reaction proceeds via an intermediate ^165^Dy isotope (t_1/2_ = 2.33 h) with a neutron capture cross-section of 3900 barns (Voorde et al. 2019). The generator-like approach allows for an accumulation of ^166^Ho to accrue over time due to the significantly longer half-life of ^166^Dy compared to ^166^Ho, and selective elution of the desired radionuclide is facile (Vosoughi et al. 2017a, b), typically utilising a metal-free HPLC system with cation-exchange columns and pH-adjusted α-HIBA as the eluent (Dadachova et al. 1994; Lahiri et al. 2004). This allows for ^166^Ho to be generated on-demand without access to a nuclear reactor and alleviates issues that would otherwise arise regarding distribution from remote production sites. The α-HIBA acts as a complexing agent, and owing to the smaller ionic radius and larger charge density of the ^166^Ho due to lanthanoid contraction, a more thermodynamically stable complex is formed compared to the analogous ^166^Dy-complex, and the ^166^Ho-complex is removed from the column first (Voorde et al. 2019; Dadachova et al. 1994). Having been separated from the ^166^Dy-complex, the ^166^Ho is demetallated from the α-HIBA chelator using acidic chloride solutions and a subsequent cation-exchange separation from α-HIBA gives ^166^Ho in a carrier-free formulation in solution with a radiochemical yield of > 95% and very low breakthrough of ^166^Dy (< 0.1%) (Dadachova et al. 1994). This separation method had a separation factor of ~ 10^3^ between ^166^Ho and ^166^Dy which was achieved in under 2 h (Dadachova et al. 1994). Similar results were reported in another study, but with essentially no ^166^Dy breakthrough, which was attributed to the use of an Aminex A7 column compared to the Dowex 50 exchangers which proved ineffective at separating the ^166^Ho from the target material (Lahiri et al. 2004).

Other chromatographic paradigms have been employed for ^166^Ho separation from ^166^Dy post-production with high radionuclidic purities being reported (Vosoughi et al. 2017b). Extraction chromatography with Eichrom LN2 resin (containing 2-ethylhexylphosphonic acid mono-2-ethylhexyl ester, HEH[EHP]) as the stationary phase extractant was used with an eluent of 1.5 M nitric acid at a temperature of 25 °C (Vosoughi et al. 2017b). This study incorporated a pre-washing phase using 0.1 M nitric acid for the removal of impurities before subsequent extraction (Vosoughi et al. 2017b). In a similar exploitation of chemical properties using α-HIBA in previous investigations, complexation of ^166^Ho over ^166^Dy is achieved due to a more thermodynamically stable chelate being formed due to holmium’s smaller ionic radius and consequently higher charge density. A flow rate of 1.5 mL/min was used to give quantitative separation in 1.5 h with a resultant no-carrier-added formulation of [^166^Ho]HoCl_3_ being isolated with radionuclidic purity of > 99.9%, a high separation yield of 76% and a radiochemical purity of > 99% (Vosoughi et al. 2017b). The ^166^Ho isolated from this production route was of high specific activity and suitable for radiolabelling purposes and the production yield was sufficient for clinical applications (Vosoughi et al. 2017b). Another methodology using a similar Eichrom Ln SPS resin (containing di-(2-ethylhexyl)phosphoric acid HDEHP) as the stationary phase extractant has also been used to significant effect for the same separation purposes (Monroy-Guzman et al. 2015; Monroy-Guzman and Jaime 2015a). This method of separation involved a multi-step process: Irradiation of the nitrate salt of ^164^Dy, formation of the parent/daughter radionuclide pair ^166^Dy/^166^Ho via dissolution of the salt target in 0.15 M nitric acid, adsorption onto the Eichrom Ln SPS resin-loaded chromatographic column, desorption and elution of ^166^Dy with 1.5 M nitric acid, then desorption of ^166^Ho with 3 M nitric acid followed by precipitation of the ^166^Ho(OH)_3_ salt through addition of NaOH to the ^166^Ho eluate, then finally re-dissolution in 0.1 M HCl to afford the desired [^166^Ho]HoCl_3_ formulation (Monroy-Guzman et al. 2015). Radionuclidic purities of > 99.9% were attained using this method with the added advantage of a relatively short separation time of 20–25 min, both of which are ideal for subsequent radiolabelling and clinical applications (Monroy-Guzman et al. 2015). The ^166^Ho produced via this method is carrier-free, and was separated with 100% efficiency from the target material and parent isotope (Monroy-Guzman et al. 2015; Monroy-Guzman and Jaime 2015a).

Electrophoresis or ion-chromatography have also been suggested as a means of separation of the ^166^Dy/^166^Ho pair to afford no-carrier-added ^166^Ho, but has not resulted in a formulation of the desired radionuclide with the appropriate activity yield or radionuclidic purity necessary for radiolabelling or further biomedical applications, as only partial separation being feasible (Dadachova et al. 1995). The target material used was Dy_2_O_3_ and the same nuclear reaction as the previous methods was employed, however the separation utilised HDEHP or tri-butyl phosphate (TBP) as the stationary chromatographic phase with elution using mobile phases of nitric acid that were relatively higher in concentration than other methods (3–12 M HNO_3_) (Dadachova et al. 1995). In the same study, electrophoresis with α-HIBA as the chelating agent for ^166^Ho was investigated, again with only partial separation being possible (Dadachova et al. 1995).

## ***Samarium: ***^***153***^***Sm***

**Fig. 6:**
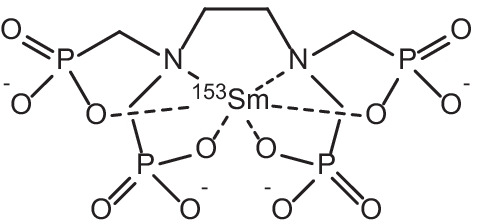
Molecular Structure of [^153^Sm]Sm-EDTMP

Samarium-153 is an attractive example of a *β*^−^-emitter with appropriate γ-emissions (energy = 103 keV, 28%) suitable for imaging in conjunction with therapy (Tan et al. 2020). Typical *β*^−^-particles emitted by this radionuclide have average energies of 0.23 MeV, with three main emission energies of 808 (18%), 705 (50%) and 635 (32%) keV respectively (Tan et al. 2020; Bombardieri et al. 2018). The combination of *β*^−^-particle emission and γ-emission in the one radionuclide has garnered attention for theranostic applications due to appropriate tissue penetration of medium-energy *β*^−^-particles (average range of 0.5 mm, and an effective range of 2–3 mm) for targeted treatment, with concurrent γ-imaging capabilities by means of SPECT or γ-scintigraphy (Voorde et al. 2019; Bombardieri et al. 2018; Sun et al. 1996). With a half-life appropriate for radionuclide therapy (t_1/2_ = 46.3 h), it has gained considerable attention due to its widespread use in the clinic as a bone pain palliation agent in patients with painful bone metastases arising as complications from various cancers (Das and Banerjee 2017; Pillai 2010; Anderson and Nuñez 2007; Jong et al. 2016; Fricker 2006). Incorporation of ^153^Sm into the ethylenediaminetetra(methylene phosphonate) (EDTMP) ligand (see Fig. 6) has proved especially efficacious for such applications, due to the targeted nature of its selective uptake into the bone matrix, particularly the new bone matrix formed by osteoblasts, and was approved by the US FDA in 1997 (Pillai 2010; Anderson and Nuñez 2007; Jong et al. 2016; Fricker 2006; Goeckeler et al. 1987; Lattimer et al. 1990; Atkins 1998). ^153^Sm complexes for use in treating bone metastases have the advantage of being capable of both imaging the affected bone sites by means of a radionuclide bone scan, and providing efficacious *β*^−^-therapy for these cancer metastases, hereby fulfilling the theranostic concept of joint diagnostics and therapy (Quadramet^®^. 2017; Sartor et al. 2004; Morris et al. 2009). As such, with bone pain metastases being fairly common complications in patients with a variety of different types of cancer (> 50% of cancer patients), ^153^Sm has found application in the treatment of a wide range of bone metastases arising as complications from a number of different types of cancer (Voorde et al. 2019). Most significantly, 80% of breast, prostate and lung cancer patients experience painful bone metastases, with reductions in bone pain being essential to patient recovery, for which the administration of [^153^Sm]Sm-EDTMP has been reported as greatly efficacious (Voorde et al. 2019; Eary et al. 1993; Silberstein 2005; Kolesnikov-Gauthier et al. 2018). Reduction of bone-metastasis-related pain after administration of [^153^Sm]Sm-EDTMP (37 MBq/kg standard dose) has been shown to occur in 55–70% of patients in certain studies (Eary et al. 1993; Silberstein 2005; El-Amm and Aragon-Ching 2016). A recent study has also shown that [^153^Sm]Sm-EDTMP was equally safe and effective as a bone pain palliation agent when compared to its ^177^Lu-labelled analogue (Taheri et al. 2018). Both radiopharmaceuticals were compared in a double-blind study for their efficacy and safety when administered for the amelioration of commonly encountered symptoms of bone metastases in cancer patients. No difference was observed between groups regarding pain alleviation, with both groups experiencing significantly reduced pain from week 2 onwards and lasting for 12 weeks post-treatment (Taheri et al. 2018). Efficacious bone pain palliation results utilising ^153^Sm have been echoed by the observation that 67% of patients with painful bone metastases have displayed overall therapeutic bone stabilisation/regression after only one dose of [^153^Sm]Sm-EDTMP (Elzahry et al. 2018).

**Fig. 7:**
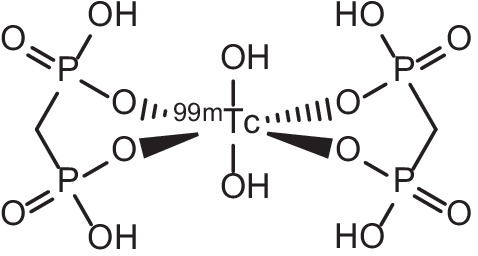
Molecular structure of bone scan agent [^99m^Tc]Tc-methylenediphosphonate

Focus on the use of ^153^Sm for such bone-metastasis applications has also been due to advantages it has over other radionuclides used for bone palliation like ^89^Sr, such as shorter physical half-life resulting in lower dose burdens to patients, lower *β*^−^-particle energy, more efficient delivery of radiation, fast bioelimination from the body and lower myelotoxicity (Tan et al. 2020; Pillai 2010; Serafini et al. 1998; Turner et al. 1989). Furthermore, the low probability of a γ-emission from ^89^Sr (909 keV, 9.555 × 10^–5^%) (Schima 1998) has led clinicians and researchers to resort to bremsstrahlung imaging or complementary radiotracer matches in order to image or monitor the administration of ^89^Sr (Qaim et al. 2018), which has led to ^153^Sm garnering attention as an alternative due to its γ-emissions being of significantly higher probability and suitable for SPECT and γ-scintigraphy (Tan et al. 2020). To this end, the γ-emission from ^153^Sm is ideally suited for high spatial resolution imaging with low signal-to-noise ratios which can be taken at different stages during treatment, with the added advantage of allowing for evaluation of radioactive leakage to other organs after each bone palliation treatment (Production 2021). Imaging of ^153^Sm for the purposes of monitoring bone palliation therapy progression, radiopharmaceutical biodistribution or dosimetry measurements is performed using either a gamma camera or a SPECT/CT scanner equipped with high resolution, low-energy collimators (Tan et al. 2020). Images attained from ^153^Sm bone scans have been compared with bone scan images that used the more common [^99m^Tc]Tc-methylenediphosphonate (^99m^Tc-MDP, see Fig. 7), and the results have been comparable in image quality and utility (Anderson and Nuñez 2007; Tripathi et al. 2006; Ramachandran et al. 2011). Comparable performance outcomes of this nature, in conjunction with its combined therapeutic and imaging efficacy, have served to reinforce the suitability and utility of ^153^Sm as a theranostic cancer agent.

Researchers and clinicians have also investigated the potential for expanding the remit of ^153^Sm radiopharmaceuticals to include the treatment of other types of cancer, due to its suitable decay characteristics for other cancer treatment applications, affordability over other radionuclides and less complicated mode of production (Tan et al. 2020). Theranostic treatment regimens for liver tumours have been proposed using ^153^Sm-labelled microparticles for transarterial radioembolization with post-procedural imaging, and an experimental study concluded that the ^153^Sm microparticles exhibited superior characteristics and performance compared to analogous ^90^Y microspheres (Hashikin et al. 2015). While the results are encouraging, dosimetry studies are imperative for ascertaining the correct amount of ^153^Sm activity required to achieve equivalent tumour doses and commensurate results compared to the readily used and commercially available ^90^Y microspheres (Tan et al. 2020). Progress was made in this regard, as a Monte Carlo simulation study was conducted to ascertain organ dose levels from hepatic radioembolization procedures using different radionuclides (Hashikin et al. 2016). The study reported that tumour dose equivalents for an activity of 1.82 GBq ^90^Y could be achieved with an estimated 4.44 GBq of ^153^Sm (~ 2.4 times the activity) (Hashikin et al. 2016). In light of these results, more investigation into specific dosimetry requirements, in vivo studies of pharmacokinetics, biodistribution and stability are needed for progress to be made in this regard (Tan et al. 2020).

Moreover, the potential for ^153^Sm to be incorporated into treatment for spinal cancer and metastases has been explored (Donanzam et al. 2013; Montaño and Campos 2019). Added advantages such as cost-effectiveness, ease of production, theranostic properties and availability have led ^153^Sm to be regarded as a potential radionuclide for these applications, with the most useful characteristic being its ability to be imaged as well as provide the appropriate *β*^−^-therapy. ^153^Sm was investigated as a potential neutron-activated radionuclide for incorporation into calcium phosphate for a biomaterial-based treatment for metastases of the spine by means of a radioactive bone cement applied to the vertebrae, hereby combining vertebroplasty and radiotherapy (Donanzam et al. 2013). The biomaterial was a calcium phosphate bone cement which was synthesised with stable Sm using the sol–gel route for calcium phosphate synthesis. The resulting bone cement (containing stable Sm) underwent neutron activation, which produced a radioactive ^153^Sm-infused bone cement that was found to have activities of ^153^Sm on the order of ~ 33 MBq/mg, which is promising for further applications in radiovertebroplasty (Donanzam et al. 2013). However, as with other approaches to radioactive bone cement (Tan et al. 2020; Hirsch et al. 2008), further clinical research and data must be collected before this relatively novel approach to spinal cancer and metastasis treatment is deemed appropriate for widespread clinical application. A similar approach was employed in another study with polymethylmethacrylate (PMMA) and hydroxyapatite (HA) as the bone cement, with results being similarly promising, however it was noted that further study regarding radiotoxicity, cytotoxicity, dosimetry, radiobiology and performance in the clinic must be conducted for further progress to be made (Montaño and Campos 2019).

Production of ^153^Sm has typically been achieved in carrier-added form by means of neutron irradiation of both natural and enriched Sm_2_O_3_ targets in a nuclear reactor, with the purity of the resulting ^153^Sm depending significantly on the enrichment of the target material (Voorde et al. 2019). These methods exploit the ^152^Sm(n,γ)^153^Sm nuclear reaction which has a relatively high neutron capture cross section of 206 barns (Tan et al. 2020). Only medium neutron fluxes are required due to the relatively high neutron capture cross-section of the reaction (Das and Pillai 2013). Disadvantages encountered during the production process for ^153^Sm have included its relatively short half-life, making production and transportation logistics challenging; the carrier-added form of the product radionuclide; the long irradiation times required for sufficient activities and yields to be produced; and the propensity for long-lived radioactive impurities to be produced from side nuclear reactions during bombardment (Voorde et al. 2019).

As can be expected, the use of natural Sm targets (3.1% ^144^Sm, 15% ^147^Sm, 11.2% ^148^Sm, 13.8% ^149^Sm, 7.4% ^150^Sm, 26.7% ^152^Sm, and 22.8% ^154^Sm) results in a product with significant impurities, the most significant of which are the long-lived ^145^Sm (t_1/2_ = 340 d), ^151^Sm (t_1/2_ = 88.8 y) and ^155^Eu (t_1/2_ = 4.76 y), and is therefore generally deemed not ideal for the production of high radiopurity and specific activity ^153^Sm (Islami-Rad et al. 2011; Chakravarty et al. 2018; Kalef-Ezra et al. 2015; Ramamoorthy et al. 2002). Despite this, progress has been made in the area of post-purification after irradiation of natural Sm_2_O_3_ targets by means of separating the desired ^153^Sm from longer-lived Eu contaminants using ion reduction, ion exchange and solvent extraction methods (Islami-Rad et al. 2011). Of these methods, the ion exchange approach has allowed for the isolation of ^153^Sm from such impurities with recovery yields of > 66% and purities > 99.8% (Islami-Rad et al. 2011). Separation has also been achieved using electrochemical means, with ^153^Sm recovery yields > 85% and acceptable radiopurities suitable for some clinical applications, exemplified by the incorporation of the purified ^153^Sm obtained into [^153^Sm]Sm-EDTMP formulations with radiolabelling yields of > 98% and radiochemical purities of > 99% (Chakravarty et al. 2018). From natural Sm targets, activities of ~ 32 GBq after electrochemical purification of a ~ 37 GBq activity batch have been obtained with the ^154^Eu and ^156^Eu contaminants not being detected, implying high radiopurity suitable for [^153^Sm]Sm-EDTMP radiolabelling (Chakravarty et al. 2018). Studies comparing the suitability of ^153^Sm produced by either natural or enriched Sm targets have generally concurred that enriched [^152^Sm]Sm_2_O_3_ targets result in higher overall activities and specific activities, however it has been noted that enriched [^152^Sm]Sm_2_O_3_ is considerably more expensive (Chakravarty et al. 2018; Ramamoorthy et al. 2002). This has prompted some researchers to focus on natural Sm targets and purification methods over purchasing enriched Sm targets (Islami-Rad et al. 2011). Additionally, it has also been noted in certain studies that, for the bone pain palliation purposes for which ^153^Sm is typically used, sufficient specific activities can be achieved using natural Sm targets (Pillai 2010; Das and Pillai 2013; Ramamoorthy et al. 2002).

For the requirements of targeted radionuclide therapy and further clinical applications, higher specific activities are needed particularly for radiolabelling purposes such as incorporation of ^153^Sm into antibodies, peptides or other targeting vectors/bifunctional chelators (Das and Pillai 2013). Obtaining sufficient specific activities for the aforementioned purposes generally requires enriched ^152^Sm targets and long irradiation times on the order of days to ensure a high yield of the required radionuclide (Voorde et al. 2019). By using an enriched [^152^Sm]Sm_2_O_3_ target with neutron irradiation at 2–5 × 10^13^ n/cm^2^/s for a period of 3 to 7 days, one study reported specific activities of 44 GBq/mg ^153^Sm, which was determined to be approximately 4 times higher than the specific activities obtained using natural Sm_2_O_3_ targets (Ramamoorthy et al. 2002). These results are echoed elsewhere with approximately 4 times higher activities of ^153^Sm being produced using enriched targets compared to natural targets, using 10 g target sizes and similar neutron irradiation beam energies and irradiation times (Chakravarty et al. 2018).

Forays into the subsequent purification of ^153^Sm produced from isotopically enriched targets has mirrored those of natural Sm targets, as some impurities arising from the nuclear reaction of the enriched target are common to the natural target, namely the long-lived ^154^Eu contaminant (t_1/2_ = 8.6 y). Importantly, the use of enriched [^152^Sm]Sm_2_O_3_ markedly reduces the side-production of ^155^Eu and ^156^Eu, but the presence of the ^154^Eu contaminant in the ^153^Sm product is very similar to the level observed using natural Sm_2_O_3_ (Ramamoorthy et al. 2002). This is due to the ^153^Eu(n,γ)^154^Eu side reaction that has a neutron capture cross section higher than that of the desired ^152^Sm(n,γ)^153^Sm reaction (312 vs. 206 barns) (Voorde et al. 2019). Such an impurity has the potential to pose issues in the clinic, as patients can only be administered 0.093 kBq of ^154^Eu per MBq of ^153^Sm during treatment (Kalef-Ezra et al. 2015; Bourgeois et al. 2011). As a consequence of this regulation, production routes for ^153^Sm using either natural or enriched targets must include measures to limit the ingrowth of ^154^Eu by either tuning their irradiation parameters to minimise the cross section of the ^153^Eu(n,γ)^154^Eu nuclear reaction and maximise the production yield of ^153^Sm, or incorporating a post-irradiation purification process (Voorde et al. 2019). Due to Eu impurities being a significant issue for ^153^Sm production from both natural and enriched targets, purification measures for ^153^Sm from both types of target are similar in their conception. Removal of these impurities from the desired product have been investigated using ion-exchange chromatography, electrochemical separation, and solvent extraction. Solvent extraction and ion-chromatographic methods of purification are more conventional, while electrochemical separation has gained traction recently (Voorde et al. 2019; Chakravarty et al. 2018). The same study that reported success with purification of ^153^Sm from natural targets using electrochemical separation also compared the same method of purification with enriched targets after irradiation (Chakravarty et al. 2018). The method involves electro-amalgamation, during which the Eu^3+^ impurities are reduced to their divalent state by means of a mercury pool cathode in an electrolytic cell. The separation occurs by way of dissolution of the Sm_2_O_3_ target (natural or enriched) in 0.1 M HCl, then transfer of the dissolved target to the separation solution of 0.15 M lithium citrate (which assists in the prevention of hydroxide precipitation) in the electrolytic cell. The electrolytic process, using a constant applied voltage of 6 V, reduces the Eu^3+^ after which transfer of the reduced Eu^2+^ ions to the mercury cathode occurs quickly, leaving behind an electrolyte solution of ^153^Sm after the mercury cathode is drained and filtered off (Chakravarty et al. 2018). This method has displayed its feasibility and applicability for purification of both natural and enriched targets, with > 85% purified ^153^Sm recovery for both target types in addition to high radiolabelling yields (> 98%) and radiopurities (Chakravarty et al. 2018). The loss of 10–15% ^153^Sm from the recovery was attributed to the reduction of Sm^3+^ to its less stable divalent form Sm^2+^ and can be amalgamated into the mercury cathode in a similar way to the Eu impurities (Chakravarty et al. 2018). This procedure was found to be suitable for large-scale ^153^Sm purification and the resultant ^153^Sm was deemed appropriate for radiolabelling purposes (Chakravarty et al. 2018). Other methods involving solvent extraction or ion exchange chromatography have been employed to exploit the differences in coordination behaviour of Eu and Sm (Islami-Rad et al. 2011; Jelinek et al. 2008, 2007; Schwantes et al. 2008; Peppard et al. 1962). An ion exchange chromatography method, developed in a similar way to that of the previously discussed ^166^Ho purification via cation-exchange HPLC, involves preferential complexation of the Eu impurities with α-HIBA and elution prior to Sm recovery. The method consists of target dissolution in 1 M HCl and subsequent separation on a Dowex-50 W cation-exchange resin with 0.2 M α-HIBA pH-adjusted to 4.8 as the mobile phase with a flow rate of 1 mL/cm^2^/min (Islami-Rad et al. 2011). In a similar manner to the separation of ^166^Ho and ^166^Dy, the smaller ionic radius and higher charge density resulting from the lanthanoid contraction leads to a preferential coordination of Eu by α-HIBA due to higher thermodynamic stability and a lower affinity for the stationary phase, and consequently the Eu elutes first, followed by the selective elution of the purified ^153^Sm. Recoveries of ^153^Sm were not as high as those reported for electrochemical separation (> 66% vs. > 85% respectively), but radiopurity was > 99.8% (Islami-Rad et al. 2011). Improvements in recovery yields and purities of the isolated ^153^Sm have been achieved by the inclusion of a Eu^3+^ reduction step prior to solvent extraction or ion exchange chromatographic separation. Reducing the contaminant Eu^3+^ to its divalent state allows for the leveraging of the significantly different chemical properties and separation/extraction behaviour exhibited by Eu^2+^ in comparison to Sm^3+^. The additional electron present in the 4*f* orbital of Eu^2+^ (4*f*^7^) results in an overall decrease in charge density which arises from the increase in the atom’s ionic radius, and hence imparts different chelation and separation kinetics compared to Sm^3+^. Certain ion exchange chromatography and solvent extraction methods have incorporated a prior reduction step into procedures that use HDEHP, as the extractant, which allows for selective chelation of Sm^3+^ ions while leaving behind the Eu^2+^ impurities (Jelinek et al. 2008, 2007; Schwantes et al. 2008; Peppard et al. 1962). The selective reduction of Eu^2+^ prior to separation results in a much lower affinity for the chosen extractant resin, and thus the Eu^2+^ is eluted first, with concentrated HCl being used subsequently for desorption and final elution of desired Sm (Jelinek et al. 2008, 2007). However, it has been noted that back-oxidation of Eu^2+^ to Eu^3+^ caused by dissolved oxygen in the system or by photooxidation is an issue with such approaches.

A recent study has also investigated the potential of utilising a quaternary ammonium ionic liquid, Aliquat 336 nitrate, for the selective extraction and purification of high purity medical ^153^Sm (Voorde et al. 2018). The study used stable isotopes of Sm and Eu, with the view that the results obtained regarding the feasibility of the method would be indicative of the results to be expected when using their radioactive counterparts. The procedure involved an aqueous feed solution of both Sm and Eu (each 1 g/L) with either a high nitrate or chloride concentration (using inert salts of NO_3_^−^ or Cl^−^ respectively) which was mixed with a large excess of Zn granules to selectively reduce the Eu^3+^ to Eu^2+^. The aqueous phase was then added to the organic phase containing Aliquat 336 nitrate and both were purged with nitrogen to prevent dissolved or atmospheric oxygen from back-oxidising the Eu^2+^. The phases were then mixed to facilitate extraction, centrifuged to allow swift phase disentanglement, and the aqueous phase was separated and analysed by inductively coupled plasma optical emission spectrometry (ICP-OES) for Sm and Eu concentrations and separation factors were calculated (Voorde et al. 2018). A subsequent back-extraction of Sm from the ionic liquid phase by diluting the aqueous phase with water gave the final purified product (Voorde et al. 2018). Results from a battery of experiments with varied mixing times and temperatures concluded that Sm^3+^ was efficiently extracted into the Aliquat 336 nitrate phase much more readily than Eu^2+^ when nitrate aqueous media are used, leaving behind the Eu^2+^ in the aqueous phase where it remains sufficiently stable (Voorde et al. 2018). This is due to the fact that Ln^3+^ ions have been shown to form stable complexes with bidentate nitrate ligands, while Ln^2+^ ions (or any other M^2+^ ions) are unable to do so (Vander Hoogerstraete and Binnemans 2014; Larsson and Binnemans 2015, 2017). High nitrate salt concentrations in the aqueous phase allowed for the Sm^3+^ ions to be salted out into the ionic liquid more readily, due to changes in ion hydration and activity that allowed for bidentate binding of Sm^3+^ by the nitrate anions, as evidenced by poorer separation factors when high chloride concentrations were used in the aqueous feed solution due to Cl^−^ ions having higher hydration energies (Voorde et al. 2019). Furthermore, it was also found that both Eu^2+^ and Zn^2+^ from the chemical reduction could be simultaneously removed from the desired Sm if a high nitrate aqueous phase was used rather than chloride, due to the similar extraction behaviours of Eu^2+^ and Zn^2+^ (Voorde et al. 2018). High separation factors were achieved by this method in a short time frame. While results for this approach appear promising, further method optimisation is required before its applicability can be successfully translated to large scale ^153^Sm production for clinical purposes.

The study also investigated the efficacy of adding a size selective extraction agent (0.05 M dicyclohexano-18-crown-6, DCH18C6) to the ionic liquid phase for selective chelation of Eu^2+^ in a similar manner to methods used for Sr^2+^ chelation. This modified approach resulted in poorer separation factors regardless of the anion used for high salt concentration in the aqueous feed solution (Voorde et al. 2018).

Furthermore, it has been suggested that improvements could be made to refine the procedure (Voorde et al. 2019, 2018). The introduction of Zn^2+^ contaminants from the chemical reduction process could be avoided by using electrolysis for reduction of Eu^3+^, and cation exchange chromatography could be used for removal of remaining salts and isolation of Sm after back extraction, after which the purified Sm solution could be reduced to dryness and the Sm redissolved in an appropriate radiolabelling solution (Voorde et al. 2019). Precipitation via hydrolysis of Sm in an alkaline medium followed by redissolution has also been proffered as possible alternative (Voorde et al. 2019). These approaches still require further exploration to determine their efficacy.

**Fig. 8:**
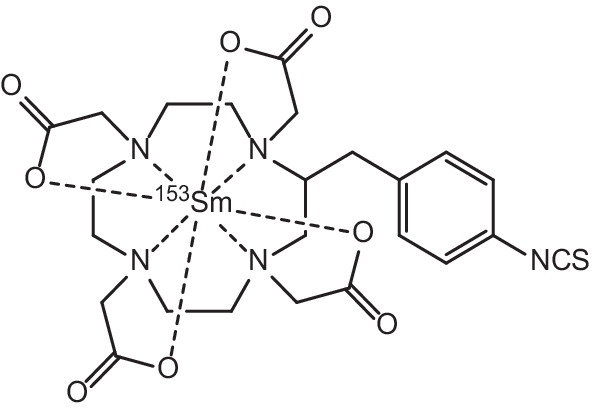
Molecular structure of [^153^Sm]Sm-*p*-SCN-Bn-DOTA

While focus on more conventional post-irradiation separation and purification of ^153^Sm has been significant, investigation into new ways of increasing the specific activity has also gained traction. In a recent novel approach, a proof-of-concept method was demonstrated for the production of very high specific activity ^153^Sm by using neutron bombardment in a high flux reactor in tandem with off-line mass separation (Voorde et al. 2021). This method involved irradiating highly enriched [^152^Sm]Sm(NO_3_)_3_ targets converted from [^152^Sm]Sm_2_O_3_ (98.7%) with neutron beam energies of 2.0–2.5 × 10^14^ n/cm^2^/s, and subsequent mass separation with laser resonance enhanced ionisation to dramatically increase the specific activity of the product radionuclide (Voorde et al. 2021). Mass separation efficiencies achieved were 4.5% on average with a maximum of 12.7%, and the ^153^Sm underwent radiochemical processing and post-purification using DGA extraction chromatography, ion exchange chromatography with α-HIBA and extraction chromatography with LN3 extraction resin to give the final [^153^Sm]SmCl_3_ formulation that was deemed suitable for radiolabelling. Specific activities as high as 1.87 TBq/mg were achieved at the time of mass separation collection after radiochemical processing. Near-quantitative yields were achieved for the radiolabelling of S-2-(4-isothiocyanatobenzyl)-1,4,7,10-tetraazacyclododecane tetraacetic acid (*p*-SCN-Bn-DOTA, see Fig. 8) and the method demonstrated the potential for TBq activites of clinical-grade ^153^Sm to be produced for radiolabelling purposes (Voorde et al. 2021). This approach is quite new, and while results are promising, further optimisation of the mass separation protocol to increase the separation efficiency and greater availability of high flux neutron reactors are required before widespread application of this method is feasible.

## ***Erbium: ***^***169***^***Er, ***^***165***^***Er***

### ^*169*^*Er*

With the widespread clinical application of ^177^Lu as a higher-energy *β*^*−*^-emitter for the treatment of various tumours, including neuroendocrine tumours (Strosberg et al. 2017), and prostate cancer (Das et al. 2016), interest has also been directed towards the search for alternative *β*^*−*^-emitters of medium or lower-energy and shorter tissue penetration lengths. Radionuclides exhibiting these characteristics have been suggested to be better suited to targeting smaller or more localised metastases that have not adequately responded to higher-energy *β*^*−*^-therapy using radionuclides such as ^177^Lu. Shorter tissue penetration lengths and lower-energy *β*^*−*^-emissions result in greater tumour-to-normal-tissue radiation dose absorption, far less collateral damage to healthy tissue surrounding the tumour site while also reducing the radiation dose burden to patients, which allow for a more personalised approach to cancer treatment based on individual patient circumstances and a tailored approach to cancer therapy which can greatly improve patient prognoses (Uusijärvi et al. 2006; Talip et al. 2021). As such, focus on the production of radionuclides that fit these criteria has been directed towards ^169^Er as a potential alternative to ^177^Lu due to its “softer” decay characteristics, particularly regarding bone pain palliation applications as ^177^Lu can cause bone marrow suppression (Talip et al. 2021; Kratochwil et al. 2019; Farahati et al. 2017; Bouchet et al. 2000). ^169^Er fulfils the aforementioned characteristics, with an acceptable half-life (*t*_1/2_ = 9.4 d) for transportation requirements and therapeutic applications, maximum *β*^*−*^-emission energies of approximately 350 keV and mean tissue penetration ranges of 0.2–0.3 mm, and with essentially no γ-emissions, establishing its advantages over higher-energy *β*^*−*^-emitters with accompanying γ-emissions which can increase patient dose burden and dosimetry issues (Uusijärvi et al. 2006). To date, ^169^Er has been primarily used for radiation synovectomy of smaller joints such as those in the fingers (Knut 2015; Karavida and Notopoulos 2010), while it has also been considered for applications in bone pain palliation of skeletal metastases arising from lung, breast or prostate cancer (Bouchet et al. 2000). The seemingly limited range of applications for this radionuclide has been hamstrung by insufficient production methods that can only provide ^169^Er in carrier-added form and with low specific activity. Despite only low specific activities being able to be achieved with current production methods, these specific activities have been deemed appropriate for radiation synovectomy, where ^169^Er is used in a citrate colloid form or in hydroxyapatite (HA) form, and lower specific activities are suitable for therapeutic use (Farahati et al. 2017; Chakraborty et al. 2014). However, the attractive radionuclide characteristics exhibited by ^169^Er have led to suggestions of targeted RIT and radionuclide therapy applications through incorporation of ^169^Er into tumour receptor, antigen-targeting or bifunctional chelator targeting vectors, with the potential for use as a theranostic pair with ^68^ Ga or, more significantly, ^44^Sc due to its similar chelation kinetics and ability to form stable complexes with bifunctional chelators already well-established in the literature (Knapp and Dash 2016; Formento-Cavaier et al. 2020). These radionuclide applications, however, require much higher specific activities of ^169^Er to be produced on a much larger scale for radiolabelling, pre-clinical and clinical use (Knapp and Dash 2016).

Production of ^169^Er has been conventionally achieved by means of neutron irradiation of both natural and highly enriched Er targets, with the level of activity being closely related to the enrichment of the target. The natural composition of Er_2_O_3_ consists of ^162^Er (0.14%), ^164^Er (1.61%), ^166^Er (33.6%), ^167^Er (22.95%), ^168^Er (26.8%) and ^170^Er (14.9%), and with a low neutron capture cross-section of approximately 2.3 barns the prospective yield from the desired ^168^Er(*n*,*γ*)^169^Er nuclear reaction is relatively poor, resulting in a low specific activity carrier-added product that is not suitable for radiolabelling purposes for which it has been suggested (Knapp and Dash 2016; Mughabghab and Mughabghab 2018). Production of carrier-added ^169^Er using natural targets has largely been superseded in favour of highly enriched [^168^Er]Er_2_O_3_ targets, due to the fact that ^163^Er, ^165^Er, ^171^Er and ^171^Tm impurities are co-produced in significant amounts when using natural targets and the low percentage of the desired ^168^Er target isotope (approximately one quarter of the natural abundance) results in low specific activity of ^169^Er (Knapp and Dash 2016; Chakravarty et al. 2014). Highly enriched [^168^Er]Er_2_O_3_ targets have allowed for a reduction in certain isotopic impurities being produced, however the low neutron capture cross section of the desired nuclear reaction still poses issues for production methods. In order to circumvent issues surrounding the low neutron capture cross section, long irradiation times of several weeks are required to achieve sufficient activity, and post-irradiation processing and purification methods have been employed to improve the specific activity of the ^169^Er product (Voorde et al. 2019; Chakravarty et al. 2014).

**Fig. 9:**
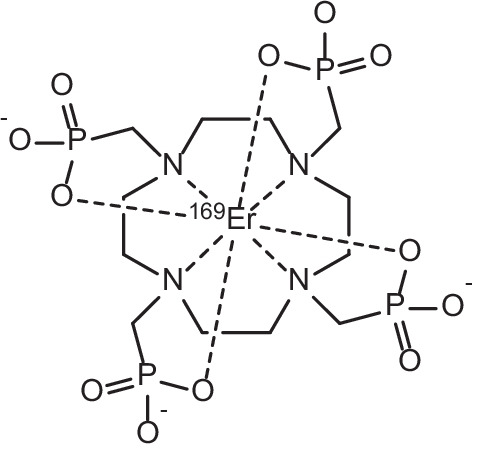
Molecular Structure of [^169^Er]Er-DOTMP

Progress towards higher specific activity formulations of ^169^Er suitable for pre-clinical and clinical purposes has been achieved with a focus on post-purification regimens due to the carrier-added nature of the conventional ^168^Er(*n*,*γ*)^169^Er neutron bombardment production method, with promising results indicated by the use of electrochemical separation and purification (Chakravarty et al. 2014). Issues noted with the use of enriched [^168^Er]Er_2_O_3_ targets and neutron bombardment have been raised regarding the co-production of ^169^Yb as an impurity, due to the presence of trace amounts of Yb in the target and the relatively high neutron capture cross section of the ^168^Yb(*n*,*γ*)^169^Yb nuclear reaction (2300 barns) (Chakravarty et al. 2014). Consequently, the removal of this impurity is paramount for the purity of the product, as well as minimising the prospect of dosimetry and dose burden issues due to the long half-life (*t*_1/2_ = 32 d) and γ-emissions characteristic of ^169^Yb decay (Chakravarty et al. 2014). Separation of ^169^Yb from the desired ^169^Er was investigated using electro-amalgamation by means of selective reduction of Yb^3+^ to Yb^2+^ and preferential electrochemical deposition onto a mercury pool cathode, similar to that reported for the separation of Eu impurities from ^153^Sm (Chakravarty et al. 2014). The requisite ^169^Er was produced in a nuclear reactor by means of the ^168^Er(*n*,*γ*)^169^Er nuclear reaction using highly enriched [^168^Er]Er_2_O_3_ target material (98.2% ^168^Er) and a thermal neutron flux of approximately 8 × 10^13^ n/cm^2^/s for an irradiation period of 3 weeks, culminating in an activity level of ~ 3.7 GBq of ^169^Er with ~ 30 MBq of ^169^Yb and a small amount of ^171^Tm as impurities at the end of bombardment. The irradiated target is then cooled and dissolved in 1 M HCl, followed by evaporation and reconstitution in de-ionised water, after which the ^169^Er/^169^Yb mixture is dissolved in 0.15 M lithium citrate buffer [to prevent the formation of Yb(OH)_3_] and pH-adjusted to 6–7 with 0.1 M NH_4_OH and stable YbCl_3_ is added as a source of carrier ions to achieve the minimum concentration of Yb^3+^ (0.6–0.8 mM) required to increase the amalgamation kinetics and ensure complete removal of ^169^Yb (Chakravarty et al. 2014). An applied potential of minimum 8 V (current = 500 mA) for a minimum of 15 min was found to be sufficient for complete removal of ^169^Yb, and the electro-amalgamation process was performed twice on the electrolyte solution. The first electro-amalgamation was shown to reduce the Yb impurities to < 0.1% with < 5% loss of ^169^Er activity, while repeating the process ensured quantitative removal of ^169^Yb, confirmed by the absence of *γ*-photopeaks corresponding to ^169^Yb in the HPGe *γ*-spectra of the purified ^169^Er solution. This method resulted in the production of radiopure ^169^Er at ~ 3.7 GBq levels with a yield of > 95% deemed suitable for radiolabelling of hydroxyapatite (HA) for radiation synovectomy and 1,4,7,10-tetraazacyclododecane-1,4,7,10-tetramethylenephosphonic acid (DOTMP, see Fig. 9) for bone pain palliation purposes, with radiolabelling yields of 98.7% and 99.1% respectively and with radiochemical purity > 99% (Chakravarty et al. 2014). Such applications, however, allow for the use of lower specific activity formulations of ^169^Er, for which the results reported were deemed appropriate. This method highlights the potential for greater specific activity ^169^Er to be produced free from isotopic impurities, however, further improvements in specific activity are required for the translation of this method of ^169^Er production and purification into theranostic applications in receptor-targeted radionuclide therapy or RIT.

Motivation to improve the specific activities attainable from the ^168^Er(*n*,*γ*)^169^Er nuclear reaction and subsequent processing has led to directed efforts to circumvent the issue of the very poor neutron capture cross-section. Investigations into methods of increasing specific activity prior to radiochemical separation and purification have proved promising, with substantial increases in specific activity reported using mass separation procedures after neutron irradiation of enriched [^168^Er]Er_2_O_3_ targets (Talip et al. 2021; Formento-Cavaier et al. 2020). Due to the issue of the carrier-added form of ^169^Er that is produced by the neutron bombardment approach, subsequent separation of ^169^Er from the ^168^Er target has proved challenging due to their chemically identical nature. Whereas conventional chemical separation techniques permit separation of impurities such as ^169^Yb and ^171^Tm due to minute differences in chelation chemistry, kinetics or electrochemical reduction properties, other separation techniques such as mass separation have been identified as promising avenues of inquiry to selectively separate target radionuclei from their carriers (Talip et al. 2021; Formento-Cavaier et al. 2020). The principle of mass separation has been employed using both electromagnetic mass separation using surface ionisation, and resonant laser ionisation (Talip et al. 2021; Formento-Cavaier et al. 2020). One reported study used offline electromagnetic isotope mass separation at the CERN-MEDICIS facility after high flux neutron irradiation of an enriched [^168^Er]Er_2_O_3_ target (~ 7 mg) for 6.5 days at a neutron beam energy of 1.3 × 10^15^ n/cm^2^/s, resulting in a calculated specific activity of 3.7 GBq/mg at the end of bombardment. After transfer of the irradiated sample to the CERN-MEDICIS facility, the calculated specific activity at the time of separation was 1.3 GBq/mg (Formento-Cavaier et al. 2020). The sample was dissolved in 1 M HNO_3_ and heated to dryness to deposit the Er sample as its nitrate salt on a graphite holder in a Re boat. The holder in positioned in the mass separator and slowly heated under vacuum to 2000–2100 °C to release the Er ions which are then mass separated and the *m/z* 169 ion beam is selectively collected by means of implantation into a Zn-coated Au foil (Formento-Cavaier et al. 2020). The mass separation procedure improved the ratio of target ^168^Er to desired ^169^Er by a factor of approximately 200, as the ~ 2000:1 ^168^Er:^169^Er ratio was decreased to ~ 10:1 with a consequent improvement in specific activity from ~ 1.3 GBq/mg to ~ 240 GBq/mg. This proof-of-principle experiment resulted in the production of a usable dose of ~ 17 MBq of ^169^Er from ~ 9 GBq present at the time of mass separation, and this study represents the first production of very high specific activity ^169^Er (Formento-Cavaier et al. 2020). Despite the improvements in specific activity exemplified by this method, the mass separation efficiency was only ~ 0.2% which is unsuitable for large-scale production purposes and requires improvement. It was posited that reduction of residual ^168^Er in the final product could be achieved by optimising the mass separator slit position to minimise the tail of the *m/z* 168 peak overlapping with the desired *m/z* 169 peak. Isotopic impurities of isobaric ^169^Yb were observed when the implanted foil was analysed at the end of bombardment, at ~ 0.2% of the atoms implanted, corresponding to an activity of ~ 10 kBq. This study did not proceed with post-purification processes, which would allow for the removal of this impurity. It was also noted that a higher ionisation efficiency would be expected using laser resonant ionisation instead of surface ionisation, and a publication detailing results from a stable Er experiment investigating this ionisation method is forthcoming (Formento-Cavaier et al. 2020). Improvements to this method have shown significant promise regarding the prospect of high specific activity ^169^Er being feasibly produced on those scales necessary for preclinical and clinical applications in receptor-targeted radionuclide therapy and theranostics, however separation efficiency remains the largest bulwark to its implementation (Formento-Cavaier et al. 2020).

Building on this proof of concept, further progress has been made regarding the use of mass separation techniques for the production of high specific-activity ^169^Er in combination with radiochemical separation procedures by employing resonant laser ionisation mass separation and chromatographic techniques for purification (Talip et al. 2021). This particular study used the newly established MEDICIS’ Laser Ion Source Assembly (MELISSA) for the offline mass separation of ^169^Er from target ^168^Er. A series of enriched [^168^Er]Er_2_O_3_ targets (7.9–14.2 mg) ware irradiated with high flux neutrons of ~ 1.1 × 10^15^ n/cm^2^/s for a period of 7 days, after which they were transferred for mass separation at CERN-MEDICIS. The irradiated samples were transferred to a Ta target container, connected to a Re ion source and heated to 2200 °C for a two-stage laser resonance ionisation using two Ti:sapphire lasers each set to an optimised wavelength for selective ^169^Er ionisation (laser 1: 24943.95 cm^−1^, laser 2: 24337.32 cm^−1^) (Talip et al. 2021; Studer et al. 2016; Lassen et al. 2017). The ^169^Er ion beam (m/z 169) arising from the resonant laser assisted ionisation was selectively mass separated using a magnetic sector field and deposited onto a Zn-coated Au foil catcher, and seven mass-separated samples were produced for subsequent radiochemical separation. The mass-separated samples were dissolved along with their Zn coated Au foil catchers in 6 M nitric acid and the resultant solution was separated on a DGA resin column by rinsing with 6 M nitric acid to selectively elute the majority of the Zn, followed by elution with 0.05 M HCl of the desired ^169^Er along with other lanthanoid contaminants (^169^Yb and ^171^Tm) that were co-produced during the neutron bombardment stage (Talip et al. 2021). This eluate was then loaded onto a Sykam macroporous cation exchange resin column and ^169^Yb was separated from ^169^Er using 0.06–0.08 M α-HIBA as the complexing agent before a third separation on LN3 resin to remove the α-HIBA and 0.02 M HCl was used to remove residual Zn. ^169^Er was subsequently eluted in 1 mL 2 M HCl and passed through a final separation on TK200 resin to ensure total removal of Zn from the final formulation which was evaporated and reconstituted in a 250 μL 0.05 M HCl solution (Talip et al. 2021). This method resulted in the production of ^169^Er activities that were four times higher than those achieved using the surface ionisation method reported previously, and the separation efficiency achieved was 0.5% when resonant laser ionisation was used (c.f. ~ 0.2% for surface ionisation) (Talip et al. 2021; Formento-Cavaier et al. 2020). Activity measurements of the samples ranged from 4.70 to 73.2 MBq and radionuclidic purities of > 99.9% were confirmed upon analysis of four of the seven samples collected. 168/169 isotope ratios were measured on the same four samples with a range of 1.60–14.62 with increases in the ratio being attributed to the tail of the mass 168 ion beam. This presented a substantial improvement over the surface ionisation method that reported ^168^Er:^169^Er ratios of ~ 10:1 (Talip et al. 2021; Formento-Cavaier et al. 2020). The activities and purities obtained were deemed suitable for the radiolabelling of PSMA-617 for subsequent use in a preliminary in vitro cell viability study, and radiochemical and chemical purities of ^169^Er were evaluated via radiolabelling of PSMA-617. [^169^Er]Er-PSMA-617 was prepared at 10 MBq/nmol concentration with a radiochemical purity > 98% and compared to the clinically-established [^177^Lu]Lu-PSMA-617 in a tumour cell viability assay using PC-3 PIP tumour cells, where results indicated reduced tumour cell viability of ~ 89% and ~ 69% at 5 MBq/mL and 10 MBq/mL respectively. These results were overshadowed by the greater reduction in cell viability displayed by ^177^Lu at the same concentration, as well as at lower concentrations of 1–2.5 MBq/mL, however it was noted that the assay could only be performed once due to the limited availability of the ^169^Er isolated from the method. Moreover, it was noted that the relatively low molar activity of the prepared radioligand likely affected tumour receptor saturation, and that further preclinical results using higher molar activity preparations of [^169^Er]Er-PSMA-617 would be needed to properly ascertain its therapeutic potential, though the initial results are promising (Talip et al. 2021). In order to obtain higher activities necessary for more extensive preclinical studies, efficiencies of the resonant laser ionisation mass separation method need to be significantly improved from 0.5% to at least 20%, commensurate with efficiency results reported for other mass separated radiolanthanoids (Talip et al. 2021; Studer et al. 2016; Kieck et al. 2019). Additionally, mass separator optimisation for high neutron flux irradiation protocols and isotope-selective laser ionisation schemes are potential developments to achieve higher ^169^Er activities and mass separation efficiencies, and longer irradiation times with minimal activity loss during separation, purification and transportation have been noted as potential ways to achieve higher ^169^Er yields on larger production scales (Talip et al. 2021).

### ^*165*^*Er*

Research efforts surrounding radiolanthanoid applications in theranostics have also focussed on potential radionuclides suitable for more targeted therapies of smaller metastases, tumours and disseminated cancers that require shorter particle path lengths and more localised energy deposition to reduce collateral damage to healthy tissues. As such, Auger electron emitters have received attention due to their favourable decay characteristics of short particle path lengths (1 nm up to ~ 5 µm) and high linear energy transfer (LET) which result in the emitted particles depositing their energy in the range of 4–26 keV/µm, suitable for highly localised targeting of important cell structures such as the nucleus or mitochondria (Kassis 2008, 2004). Auger electrons (AEs) are emitted as the result of decay by means of electron capture or internal conversion, and multiple AEs can be emitted during one nuclear decay event (Buchegger et al. 2006). These AEs are capable of damaging DNA by way of direct interaction causing double strand breaks (Martin et al. 1988), or via the formation of radicals from the hydrating water molecules surrounding the DNA macromolecule (Nikjoo et al. 1996). Consequently, Auger emitters must be highly targeted and lie in very close proximity to the important cellular structures for effective treatment (Kassis 2004; Ramogida and Orvig 2013; Bousis et al. 2010). One such radionuclide that has attracted attention as a potential Auger emitter for radionuclide therapy and theranostic applications is ^165^Er, due to its favourable half life (t_1/2_ = 10.4 h) and electron capture decay mechanism resulting in AE emissions of 5.3 keV (65.6%) and 38.4 keV (4.8%), with accompanying low-energy X-rays (average energy 48.8 keV) (Talip et al. 2021; Gracheva et al. 2020). In addition, the absence of any γ-emission during ^165^Er nuclear decay corresponds to a lower dose burden for patients (Gracheva et al. 2020). Interest in this radiolanthanoid has also been bolstered by its AE emissions having similar energies to those of ^125^I (t_1/2_ = 59.4 d), which has been studied extensively for its DNA cytotoxicity due to its AE emission properties (Gracheva et al. 2020; Yasui et al. 2001). As with ^169^Er, ^165^Er also has the potential to be paired with companion diagnostic radionuclides such as ^68^ Ga or, in particular, ^44^Sc for theranostic applications due to similarities in complexation kinetics afforded by the similar chemical properties of the Rare Earth metals (Cotton 2006; Moeller et al. 1965). Moreover, the need for pre-clinical studies to elucidate any additional therapeutic effect of Auger therapy in combination with *β*^−^-therapy through administration of both ^169^Er and ^165^Er in different ratios has been highlighted as an interesting avenue of inquiry (Talip et al. 2021).

There have been a number of nuclear reaction routes reported for the production of ^165^Er, typically using protons, deuterons or neutrons as the bombardment particles with either natural or enriched Er targets, or natural Ho targets (100% natural abundance of ^165^Ho) (Sadeghi et al. 2010; Zandi et al. 2013; Beyer et al. 2004b; Tárkányi et al. 2007, 2008a, b, c, 2009; Hermanne et al. 2013). The use of natural Er targets utilises the generator approach to produce ingrown ^165^Er after the *β*^−^-decay of ^165^Tm produced via either the ^nat^Er(p,xn)^165^Tm → ^165^Er or the ^nat^Er(d,xn)^165^Tm → ^165^Er nuclear reactions, giving the resultant ^165^Er in no-carrier-added form after separation from the target material (Tárkányi et al. 2007; Vaudon et al. 2018). These charged particle bombardment approaches on natural Er have resulted in the production of reasonably long-lived ^167^Tm (t_1/2_ = 9.25 d) and ^168^Tm (t_1/2_ = 93.1 d) impurities, and the natural abundance of the main contributing ^166^Er isotope being only ~ 33.5% has led to enriched ^166^Er targets being preferred (Vaudon et al. 2018). Furthermore recently, alpha particle bombardment has been investigated via the ^nat^Er(α,x)^165^Tm → ^165^Er route, but was deemed not practical when compared to proton and deuteron bombardment due to low yields of ^165^Tm (Aliev et al. 2021; Kormazeva et al. 2021). Enriched ^166^Er targets for the ^166^Er(p,2n)^165^Er nuclear reaction are expensive and have therefore led to shifts in research focus to ^165^Ho targets paired with proton and deuteron bombardment, with subsequent chemical separation (Tárkányi et al. 2008b, c). These production methods have been favoured due to the fact that they exhibit lower target material costs due to the 100% natural abundance of ^165^Ho, and the feasibility of the ^165^Ho(p,n)^165^Er and ^165^Ho(d,2n)^165^Er nuclear reactions which can be achieved at commercial cyclotrons already in operation using proton and deuteron beams of lower energies (< 20 MeV) (Zandi et al. 2013; Tárkányi et al. 2008b, c; Vaudon et al. 2018). Of these two nuclear reactions, the deuteron reaction has been reported to produce larger amounts of ^165^Er activity at low-energy cyclotrons, but with the trade-off of higher amounts of ^166^Ho as an impurity, with ^165^Er/^166^Ho ratios of ~ 400/1 c.f. 8/1 for proton and deuteron irradiation respectively (Tárkányi et al. 2008c; Vaudon et al. 2018). A noteworthy advantage of lower-energy irradiation beams is that only ^165^Er is produced when low-energy protons are used, however lower-energy deuteron beams can result in stable ^166^Er being produced on the ^165^Ho targets in addition to ^165^Er which gives a no-carrier-added but not carrier-free product (Tárkányi et al. 2009). In contrast, at higher energies (> 30 MeV), ^nat^Er(p,xn)^165^Tm → ^165^Er is the preferred reaction (Tárkányi et al. 2008a, 2009). Further challenges arise in the separation of ^165^Er from the ^165^Ho target, with separation factors for the Er/Ho pair being among the lowest for lanthanoid separations (~ 1.5), and greater improvements are thereby required for further practical applications of ^165^Er to be attainable. Certain separations of ^165^Er and ^165^Ho have centred around LN2 resin and nitric acid as the eluent (similar to previous methodologies for radiolanthanoid separations), however it has been reported that the reduced adsorption capacity of LN2 resin limits the amount of ^165^Er that can be successfully separated (Vaudon et al. 2018). Further investigation into optimisation of post-purification has occurred more recently, with the production of ^165^Er by means of the proton irradiation route at 8.6–16.2 MeV beam energies on ^165^Ho_2_O_3_ targets at low-energy cyclotrons (Gracheva et al. 2020). In this study, an irradiation and separation protocol for larger amounts of ^165^Ho target material (up to 200 mg ^165^Ho_2_O_3_) was developed for the isolation of ^165^Er suitable for radiolabelling and in vivo applications. The maximum cross section of ~ 180 mb for the ^165^Ho(p,n)^165^Er nuclear reaction was achieved at a beam energy of 10.3 MeV, and cross section measurements over the range of 6.7–18.2 MeV were in good agreement with those previously reported (Tárkányi et al. 2008b). Activities of ^165^Er up to 1.6 GBq were produced via irradiation of a 10 μA target pellet at the optimum beam energy of ~ 13.4 MeV (determined from shorter irradiations and yield measurements) for 8–10 h, and the co-production of ^163^Er was avoided by ensuring that an impinging energy lower than the ^165^Ho(p,3n)^163^Er threshold energy [17 MeV—TALYS-based evaluated nuclear data library 2017 (TENDL-2017)] was used. Subsequent separation of ^165^Er from the target material employed a two-column method of cation-exchange chromatography on Sykam resin using 0.08 M α-HIBA followed by extraction chromatography on a LN3 column with 0.1 M HCl for final formulation concentration. A hot cell was used in the separation module to allow for higher activities of ^165^Er to be used, and ^165^Er was separated from the target Ho and co-produced Tm impurities in 7 h and eluted in 500 µL 0.1 M HCl in its final formulation. Five separations were performed and the radionuclidic purity of the [^165^Er]ErCl_3_ product was ascertained by measuring the characteristic 47.6 and 46.7 keV X-ray energies of ^165^Er using a HPGe detector (> 99.9% ^165^Er with no other detectable radionuclide impurities) (Gracheva et al. 2020). ICP-OES was used on two of the purifications to calculate the natural Er content of the product, with ^nat^Er/^165^Er ratios measured to be 60/1 and 51/1 respectively, indicating that the product was not carrier free and consequently not suitable for DOTA-NOC radiolabelling. The presence of ^nat^Er in the final formulation was attributed to impurities in the Ho target material, as the presence of certain Tm impurities (namely ^166^Tm, ^167^Tm and ^168^Tm) formed from (p,n) reactions on ^nat^Er isotopes also suggested this. To rectify this issue, separation of natural Er impurities from the ^165^Ho_2_O_3_ target prior to irradiation was required, and a Ho target recycling procedure was developed which involved increasing the α-HIBA concentration to 0.11 M and eluting the ^165^Ho_2_O_3_ target separately after ^165^Er elution. Nitric acid (1.0 M) was added to the Ho eluate and the resultant solution was separated on another column using AG MP-50 macroporous cation exchange resin. The ^165^Ho was eluted as its nitrate salt in 40 mL 7.0 nitric acid, evaporated to dryness, and finally converted to its oxide by heating at high temperature (> 600 °C) (Gracheva et al. 2020). This recycling method recovered 845 mg of Ho target material, and subsequent irradiations of the recycled target material with post-purification to evaluate the efficacy of target recycling in removing target contaminants prior to irradiation are forthcoming. Irradiation of recycled Ho targets free from natural Er impurities holds promise for higher specific activity no-carrier-added formulations of ^165^Er to be produced, as future DOTA-NOC radiolabelling studies using ^165^Er from recycled targets are on the horizon (Gracheva et al. 2020). While this production and purification process holds promise for ^165^Er to be produced for preclinical applications, the ~ 10 h purification time results in a significant loss in activity in the final formulation due to the half life of ^165^Er (t_1/2_ = 10.4 h), which poses issues for large scale production and clinical applications which require further method development and process optimisation (Gracheva et al. 2020).

Despite the previous intimation that enriched ^166^Er targets are prohibitively expensive, work has progressed on evaluating the feasibility of both the ^166^Er(p,2n)^165^Tm → ^165^Er and the ^166^Er(d,3n)^165^Tm → ^165^Er cyclotron production methods (Sadeghi et al. 2010; Zandi et al. 2013). Calculations have been performed using the ALICE/ASH and EMPIRE-3.1 codes for nuclear reactions to evaluate the excitation functions, yields and target thicknesses that are optimum for the four routes to ^165^Er, namely ^166^Er(p,2n)^165^Tm → ^165^Er, ^nat^Er(p,x)^165^Tm → ^165^Er, ^165^Ho(p,n)^165^Er and ^165^Ho(d,2n)^165^Er, and these have been compared against experimental data obtained from other investigations (Zandi et al. 2013). From these data, it was concluded that the proton irradiation of ^166^Er for the generator production of ^165^Er from ^165^Tm afforded the highest production yield of ^165^Er in no-carrier-added form (Zandi et al. 2013). The ^166^Er(p,2n)^165^Tm → ^165^Er reaction, for optimum yield of ^165^Er, requires proton beam energies of 16–23 MeV with a maximum nuclear reaction cross section at 21 MeV, which is readily achievable at low-energy cyclotrons (18 MeV), but yield maximisation would require higher beam energies not available at such facilities (Zandi et al. 2013). For the analogous deuteron reaction on ^166^Er, a maximum cross Sect. (1523.9 mb) was calculated for deuteron beam energies of 25 MeV with an optimum range of 22–29 MeV, however the co-production of ^166^Tm (t_1/2_ = 7.7 h), ^164^Tm (t_1/2_ = 2 min) and ^163^Tm (t_1/2_ = 1.81 h) impurities posed purification issues. Furthermore, the required deuteron beam energies are higher than those available at 18 MeV commercial cyclotrons, which necessitates the use of more specialised facilities which are scarce (Sadeghi et al. 2010).

The potential for the production of ^165^Er using an on-line mass separation approach has also been suggested, with a proof-of-principle experiment performed at the ISAC Facility at TRIUMF and the resulting ^165^Er used for the successful radiolabelling of DOTA (Fiaccabrino et al. 2021). This method implanted a mass 165 ion beam from a Ta foil target into an Al implantation target, and the ^165^Er and ^165^Tm activity was retrieved via repeated evaporation and rinsing of the implantation target surface layer with 0.1 M HCl (Fiaccabrino et al. 2021). A post-purification procedure based on LN resin and HCl was used, however complete separation of ^165^Er from ^165^Tm was not achieved and the fraction with the highest percentage of ^165^Er activity (~ 17%) and lowest level of ^165^Tm impurity was selected for radiolabelling of macropa and DOTA. Macropa radiolabelling was unsuccessful (< 1%) whereas quantitative labelling of DOTA was achieved (> 99%). Despite these results, further improvements in purification are necessary before more substantial radiolabelling and preclinical studies can be conducted. Further work at the ISAC facility regarding yield measurements, radiolabelling and preclinical studies for a number of medically relevant radiolanthanoids, including ^165^Er, have been planned in the future (Fiaccabrino et al. 2021).

## ***Promethium: ***^***149***^***Pm***

**Fig. 10:**
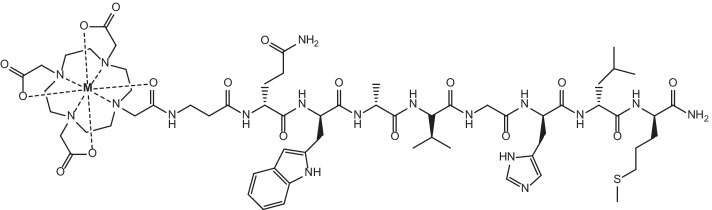
Molecular structure of lanthanoid DO3A-amide-βAla-BBN complexes, where **M** = ^149^Pm, ^153^Sm, ^177^Lu

**Fig. 11:**
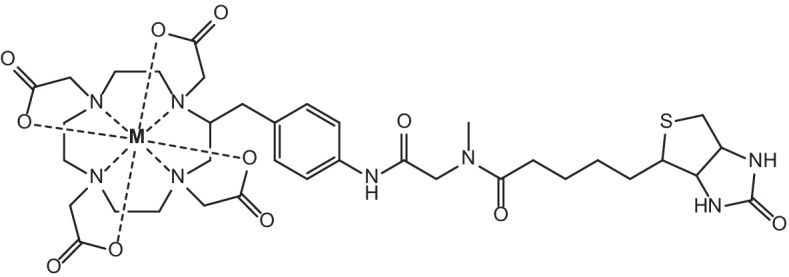
Molecular structure of lanthanoid DOTA-biotin complexes, where **M** = ^149^Pm, ^166^Ho, ^177^Lu

Of the radiolanthanoids currently in use in medical applications today, only three can be readily produced in no-carrier-added form from a different lanthanoid target material (^149^Pm, ^166^Ho and ^177^Lu) (Hu et al. 2002). ^149^Pm has been considered a promising addition to the library of theranostic radionuclides due to its favourable decay characteristics and reasonable physical half-life (t_1/2_ = 2.21 d) (Hu et al. 2002). This radiolanthanoid exhibits appropriate *β*^−^-decay characteristics (1.07 MeV (95.9%)) with an accompanying γ-emission (286 keV (2.8%)) which is appropriate for concurrent imaging in theranostic settings as well as for dosimetry and biodistribution evaluation, while also being of low enough energy to avoid dose burden complications in potential clinical applications (Hu et al. 2002; Nieschmidt et al. 1965; Bunney et al. 1960). In addition, the ability for this radionuclide to be produced in no-carrier-added form with high specific activity permits its use in RIT and receptor-targeted radionuclide therapy applications which necessitate high specific activities and radiopurity for successful radiolabelling, with preclinical studies reporting promising results for future applications (Hu et al. 2002; Lewis et al. 2004; Mohsin et al. 2006, 2011). Preclinical work for receptor-targeted radiotherapy applications has seen ^149^Pm incorporated into DOTA bombesin analogues for in vivo comparison with [^153^Sm]Sm- and [^177^Lu]Lu-DO3A-amide-βAla-BBN complexes (see Fig. 10), with close to identical behaviour being observed across the three complexes with regards to biodistribution (Hu et al. 2002). Pretargeted RIT potential has also been investigated through the preparation of [^149^Pm]Pm-, [^166^Ho]Ho- and [^177^Lu]Lu-DOTA-biotin complexes (see Fig. 11) and pretargeting them to LS174T colorectal tumours in nude mice with the CC49 scFvSA antibody fusion protein, with tumour uptake and biodistribution of the ^149^Pm complex performing commensurately with those of the ^166^Ho and ^177^Lu complexes, with favourable tumour targeting, body clearance and urinary excretion being reported (Lewis et al. 2004). Further preclinical work in LS174T human colon carcinoma xenograft nude mice models with *N*-hydroxysulfosuccinimidyl DOTA (DOTA-OSSu) and methoxy-DOTA (MeO-DOTA) complexes of ^149^Pm conjugated to the CC49 anti-TAG-72 monoclonal antibody has also reported high tumour uptake and favourable biodistribution properties for RIT applications, with MeO-DOTA affording higher conjugate stability and tumour uptake as well as lower kidney retention compared to DOTA-OSSu (Mohsin et al. 2006). Both pretargeted and conventional RIT methods for LS174T colon tumours in nude mice models have been compared for their efficacy using [^149^Pm]Pm-, [^166^Ho]Ho- and [^177^Lu]Lu-DOTA-biotin and CC49 compounds, with [^149^Pm]Pm-DOTA-biotin and [^149^Pm]Pm-CC49 compounds both considerably increasing median survival over saline controls, significantly inhibiting tumour growth and exhibiting high tumour selectivity with little to no off-target tissue toxicity (Mohsin et al. 2011). In comparison to ^166^Ho and ^177^Lu, the ^149^Pm complexes outperformed the analogous ^166^Ho complexes in median survival, median time of progression and tumour doubling time, whereas ^177^Lu was deemed more efficacious than ^149^Pm (Mohsin et al. 2011). As such, preclinical studies have reported encouraging results for the application of ^149^Pm in RIT, and have established ^149^Pm as a potential alternative to the well-established ^90^Y for therapies where shorter tissue penetration ranges, imageable γ-emissions or softer *β*^−^-energies are required (Uusijärvi et al. 2006; Mohsin et al. 2011). Radiolabelling and preclinical investigation into the in vitro stability, in vivo stability and biodistribution of aminocarboxylate and octreotide complexes of no-carrier-added ^149^Pm in comparison with the analogous ^166^Ho and ^177^Lu complexes has also been conducted (Li et al. 2003).

Production of ^149^Pm in no-carrier-added form is readily achieved by means of neutron bombardment of enriched ^148^Nd targets via the ^148^Nd(n,γ)^149^Nd nuclear reaction (σ_th_ = 2.5 barn) with subsequent *β*^−^-decay of ^149^Nd (t_1/2_ = 1.73 h) to ^149^Pm, potentially allowing for theoretical specific activities of 15 GBq/mg (Uusijärvi et al. 2006; Nieschmidt et al. 1965; Bunney et al. 1960; Abrarov and Aminova 1975). The use of highly enriched ^148^Nd targets ensures the avoidance of coproduction of the reasonably long-lived ^147^Nd (t_1/2_ = 11 d) impurity due to the comparable thermal neutron capture cross-section of the ^146^Nd(n,γ)^147^Nd nuclear reaction (σ_th_ = 1.4 barn). The desired ^149^Pm is readily obtained via chemical separation from the Nd target after irradiation using extraction chromatography based on HDEHP resin (40% by weight) loaded onto Amberchrom CG-71 inert polymeric absorbent (60% by weight) as the stationary phase, and HNO_3_ as the eluent (Monroy-Guzman et al. 2015; Ketring et al. 2003; Monroy-Guzman and Jaime 2015b). In one study, the Nd target was loaded onto the HDEHP/Amberchrom column after being dissolved in 0.15 M HCl, and the Nd was eluted first due to its slightly lower charge density and ionic radius using 0.5 M HNO_3_ after which the desired ^149^Pm was eluted using 5 M HNO_3_ (Ketring et al. 2003). The ^149^Pm fractions are then evaporated and reconstituted in a suitable solution for further applications (usually HCl) with high radiopurity. More recently, similar chromatographic methods have been employed for the separation of micro–macro component systems typically encountered when producing no-carrier-added and/or carrier free radioanthanoids (Monroy-Guzman et al. 2015; Monroy-Guzman and Jaime 2015b). HDEHP resin on an Ambercrhom CG-71 support were again used, however irradiation targets were dissolved in 0.15 M HNO_3_ for column loading and distribution coefficients and separation factors were calculated in order to optimise the separation of the desired radiolanthanoid from the bulk target material (Monroy-Guzman et al. 2015; Monroy-Guzman and Jaime 2015b). Specifically for the separation of ^149^Pm from Nd, optimum separation was achieved when 0.18 M HNO_3_ was used for elution of Nd and ^149^Pm was stripped from the column with 1.5 M HNO_3_, resulting in a separation efficiency of 98.4%. The ^149^Pm was then precipitated out from the HNO_3_ solution as ^149^Pm(OH)_3_ through addition of NaOH to the eluate, and the hydroxide was subsequently redissolved in 0.1 M HCl to give [^149^Pm]PmCl_3_ in its final carrier free formulation with a radiopurity of > 99.9% (Monroy-Guzman et al. 2015; Monroy-Guzman and Jaime 2015b).

Other irradiation methods have been investigated on natural Nd targets and cross-sections have been measured for the ^148^Nd(p,γ)^149^Pm, ^150^Nd(p,2n)^149^Nd → ^149^Pm, ^148^Nd(d,n)^149^Pm, and ^150^Nd(d,3n)^149^Pm nuclear reactions (Yang et al. 2015; Tárkányi et al. 2017; Lebeda et al. 2014, 2012; Tárkányi et al. 2014). Measured cross-sections of these reactions are considerably lower than the well-established ^148^Nd(n,γ)^149^Nd → ^149^Pm nuclear reaction, and so have not seen routine use for ^149^Pm production. Moreover, investigation into the production of ^149^Pm by photonuclear means has also been undertaken by way of the ^150^Nd(γ,n)^149^Nd → ^149^Pm nuclear reaction with multi-stage extraction chromatography techniques for separation (Dikiy et al. 2015). This method used bremsstrahlung energies of up to 12.5 MeV and natural Nd targets. While high specific activities of ^149^Pm are reportedly producible via this method on a daily basis (0.5 Ci ^149^Pm per day), the cross-section for the reaction is only 220 mb (*c.f.* 2.5 barns for ^148^Nd(n,γ)^149^Nd → ^149^Pm) and ^147^Pm impurities are also produced via the ^148^Nd(γ,n)^147^Nd → ^147^Pm side nuclear reaction which has a comparable cross-section of ~ 200 barns (Dikiy et al. 2015). It has been noted that enriched ^150^Nd targets are able to increase the daily yield to 10 Ci per day, however the requirement of a linear accelerator with electron energies of 36 MeV and currents of 260 μA has limited the overall feasibility of this irradiation method for larger scale ^149^Pm production (Dikiy et al. 2015).

## ***Praseodymium: ***^***143***^***Pr***

Another radiolanthanoid that has been identified for its theranostic potential is the moderate-energy *β*^−^-emitter ^143^Pr (Mishiro et al. 2019). As a pure *β*^−^-emitter (E_β(max)_ = 0.93 MeV, E_β(mean)_ = 0.315 MeV/decay) with a relatively long half life (t_1/2_ = 13.6 d) and the additional advantage of no-carrier-added formulations being obtainable, ^143^Pr has been proposed for use in radiolabelled antibodies for RIT (Uusijärvi et al. 2006; Vimalnath et al. 2005; Humm 1986). Selection of a radionuclide for RIT is contingent upon appropriately matching the physical half-life of the radionuclide to the biological half-life and pharmacokinetic properties of the antibody to which the radionuclide will be labelled (Humm 1986). Due to the fact that antibodies may to take up to several days to accumulate and localise at the therapy site and display slow or delayed clearance from the rest of the body (i.e. other organs or the blood), radionuclides for such therapy must have sufficiently long half-lives as not to decay too rapidly before maximum tumour uptake or localisation of the radiolabelled antibody is achieved. Moreover, the photon abundance of the candidate radionuclide is imperative, since the slower pharmacokinetic properties of antibodies and longer radionuclide half-lives necessitate longer radioimmunoconjugate exposure times for RIT patients to ensure optimal tumour uptake and body clearance. Consequently, radionuclides with higher photon abundances will result in a higher absorbed dose to the entire body of the patient as the radioimmunoconjugate is metabolised and localised over a longer period of time, whereas lower photon abundances alleviate this dose burden to normal body tissue. ^143^Pr exhibits a reasonably long half-life in conjunction with its medium-energy *β*^−^-emission and low photon abundance (p/e < 0.2) which have been deemed to be suitable decay characteristics for potential RIT applications. Furthermore, inquiry into the tumour-to-normal-tissue distribution ratios (TNDs) of potential therapeutic radionuclides has demonstrated that ^143^Pr exhibits small tumour TND values similar to those of the well-established RIT nuclide ^131^I, and higher TND values for larger tumours compared to ^131^I due to lower photon emission which reduces extraneous damage to healthy tissue during uptake and localisation (Uusijärvi et al. 2006).

Production approaches for ^143^Pr have centred around two nuclear reaction schemes (Knapp and Dash 2016; Vimalnath et al. 2005). The less desirable production route involves neutron irradiation of ^141^Pr target material (100% natural abundance) by means of the ^141^Pr(n,γ)^142^Pr nuclear reaction (σ_th_ = 11.49 barns) to produce the intermediate ^142^Pr isotope, which then undergoes a second neutron capture event via the ^142^Pr(n,γ)^143^Pr nuclear reaction, thereby exploiting the higher thermal neutron capture cross-section of the intermediate ^142^Pr (σ_th_ = 20.03 barns) (Knapp and Dash 2016). This method gives the desired ^143^Pr in carrier-added form and typically results in a significant ^142^Pr impurity being present, the amount of which is highly dependent upon the irradiation time and the cooling period post-irradiation. Additionally, the compounding of the relatively low thermal neutron capture cross sections for both nuclear reactions culminates in low production yields for this method.

The second production method involves the use of Ce targets and neutron bombardment to obtain carrier-free ^143^Pr by means of the indirect ^142^Ce(n,γ)^143^Ce → ^143^Pr nuclear reaction (σ_th_ = 0.95 barns) followed by chromatographic separation of ^143^Pr from the bulk cerium target (Vimalnath et al. 2005; Kubota 1976; Peppard et al. 1957). Both natural and enriched ^142^Ce targets have been used for this method, and as with other radionuclide production routes, the use of natural ^142^Ce targets has resulted in the by-production of impurities (Vimalnath et al. 2005). Natural cerium contains ^136^Ce (0.185%), ^138^Ce (0.251%), ^140^Ce (88.45%) and ^142^Ce (11.114%), all of which have the potential to undergo neutron bombardment and form a number of impurities in various ratios that depend upon the length of bombardment, neutron flux and cooling period. The most significant impurities formed are ^137^Ce, ^139^Ce and ^141^Ce, with ^141^Ce (t_1/2_ = 32.5 d) being a particularly significant impurity due to its longer half-life compared to the desired ^143^Pr, the high natural abundance of its parent nuclide (^140^Ce – 88.45% natural abundance) and the comparable neutron capture cross section of the parent nuclide (σ = 0.57 barns) (Vimalnath et al. 2005). ^141^Ce is also a radiolanthanoid deemed suitable for potential therapeutic purposes, and so the concurrent production of two therapeutic radionuclides necessarily increases the emitted radioactivity level of the target material which can pose handling issues prior to chromatographic separation, and so ^142^Ce enriched targets are required to minimise the potential radiation dose burden (Knapp and Dash 2016). Separation of ^143^Pr from irradiated Ce targets has been investigated by means of precipitation followed by alumina column separation or filtration (Vimalnath et al. 2005), cation exchange chromatography (Kubota 1976), as well as by extraction with dioctyl phosphoric acid (Peppard et al. 1957).

More recently, production of no-carrier-added ^143^Pr from natural Ce(NH_4_)_4_(SO_4_)_4_ and CeO_2_ target materials has been reported using neutron beam energies of ~ 1 × 10^13^ n/cm^2^/s and an irradiation period of 7 days (Vimalnath et al. 2005). At EOB, 7.4 MBq/mg and 2.3 MBq/mg activities of ^143^Ce were produced from the CeO_2_ and Ce(NH_4_)_4_(SO_4_)_4_ targets respectively, with ^139^Ce and ^141^Ce impurities present in non-negligible amounts. After bombardment, the targets were either dissolved in concentrated HNO_3_ (Ce(NH_4_)_4_(SO_4_)_4_ target) or concentrated HNO_3_ with 30% H_2_O_2_ (CeO_2_ target) and were stood for a cooling period of 5 days to allow in-growth of ^143^Pr from decaying ^143^Ce. Following this, 1 M NaBrO_3_ was added to each of the irradiated target solutions with heating at 80 °C for 10 min before the addition of saturated HIO_3_ and cooling on an ice bath to precipitate out the cerium target as Ce(IO_3_)_4_. Two final separation protocols were used to obtain ^143^Pr, using either an alumina column or filtration. For column separation, the target solution with precipitate was loaded onto an alumina stationary phase column and 30 mL 1% HIO_3_ solution was used as the eluent, followed by a subsequent column washing with 20 mL double distilled water after which the collected ^143^Pr eluate was evaporated and reconstituted as [^143^Pr]PrCl_3_ in 0.1 M HCl in its final formulation (Vimalnath et al. 2005). For filtration after precipitation, the target solution with precipitate was filtered through a G-2 sintered funnel and the ^143^Pr filtrate was evaporated and reconstituted in 0.1 M HCl as [^143^Pr]PrCl_3_ (Vimalnath et al. 2005). These separation methods culminated in the isolation of ^143^Pr activities of 0.9 MBq/mg of CeO_2_ target and 0.3 MBq/mg of Ce(NH_4_)_4_(SO_4_)_4_ target, with high resolution gamma spectrometry indicating that potential Ce isotope contamination in the final [^143^Pr]PrCl_3_ solutions was lower than the detection limit and hence would not affect the final specific activity or radiopurity. The absence of Ce in any appreciable amount was corroborated by negative phosphomolybdic acid, benzidine and H_2_O_2_/NH_3_ spot tests. The resultant no-carrier-added ^143^Pr was of high radiopurity and suitable for hydroxyapatite radiolabelling (99.5% radiolabelling efficiency) (Vimalnath et al. 2005). Despite the no-carrier-added and radiopure nature of the product, issues such as the harsh column conditions, the large amount of Ce target material to separate from the desired ^143^Pr and the volume of eluent necessitated a separation time of 3–4 h for the column approach, which in addition to the time required for evaporation and reconstitution resulted in protracted purification times which are not desirable for large scale radionuclide production. Furthermore, the Ce(IO_3_)_4_ precipitation approach resulted in excess NaBrO_3_ and NaBr being present in the final [^143^Pr]PrCl_3_ formulation, which could reportedly be removed by repeating the evaporation of the residue, however reconstitution with HCl still affords a final formulation with significant NaCl, NaI and NaBr chemical impurities which are not ideal for further radiolabelling applications and improvements to the radiochemical separation protocol are required (Vimalnath et al. 2005). Additional improvements to this approach include the use of enriched ^142^Ce target materials to reduce the co-production of isotopic impurities and improve overall activity yield, and increasing the irradiating neutron flux to mitigate the relatively low neutron capture cross-section of the ^142^Ce(n,γ)^143^Ce reaction and the long irradiation times required for sufficiently high activity of ^143^Ce to be produced. Optimising these conditions could allow for the production of up to 4–6 GBq of no-carrier-added ^143^Pr from each batch (Vimalnath et al. 2005).

Separation of target Ce from ^143^Pr has also been achieved via cation-exchange chromatographic methods (Kubota 1976). Such methods exploit the inherent differences in charge density and ionic radius of Ce^4+^ compared to trivalent ^143^Pr to separate the desired radionuclide from the target material. CeO_2_ is chosen as an ideal target material due to the Ce being tetravalent and thus more easily separable from ^143^Pr^3+^ due to a smaller ionic radius and higher charge density which result in different complexation kinetics and interactions with eluents, extraction resins or stationary phases used in cation-exchange separation. In one study, due to reported issues with the dissolution of CeO_2_ in HNO_3_ or HCl media, natural CeO_2_ was formed from the pyrolysis of natural high purity Ce_2_(CO_3_)_3_ in order to improve the solubility of the oxide in HNO_3_/H_2_O_2_ solutions (Kubota 1976). The prepared CeO_2_ target was subjected to a 7.6 × 10^12^ n/cm^2^/s neutron flux for 77 h and left for a cooling period of 1 month, after which it was readily dissolved in concentrated HNO_3_ with 30% H_2_O_2_ and the solution evaporated to dryness and reconstituted in aqueous HCl. The reconstituted residue was then loaded onto a Diaion SK-1 cation-exchange column which was washed with distilled water. Elution was performed with 0.25 M citrate solution at a flow rate of 27 mL/hr and separation of ^143^Pr from target Ce and impurities was monitored by γ-spectrometry (Kubota 1976). A second cation-exchange separation on a smaller column was performed with the ^143^Pr-containing fractions using HCl as the eluent, and the final ^143^Pr-containing fractions were combined, evaporated and reconstituted in HCl. The elution profile for the separation showed the presence of overlapping ^147^Nd and ^141^Ce impurities, each respectively arising from either impurities in the natural target material or the high natural abundance of parent ^140^Ce. These contaminants could be avoided by using an enriched ^142^CeO_2_ target material. Despite overlapping impurities, the intervening fractions free of impurities contained > 99% of the total ^143^Pr activity (1.03 mCi) with a radiopurity of > 99.99% confirmed by γ-ray spectra and *β*^−^-activity monitoring over 2 months (Kubota 1976).

Other separation methods for the Ce/Pr lanthanoid pair have also exploited the differing chemistry exhibited by Ce in its tetravalent state, namely via extraction with dioctyl phosphoric acid (Peppard et al. 1957), or more recently by using liquid–liquid extraction methodologies with ionic liquids (Gras et al. 2017). Similar to other ionic liquid extraction techniques for lanthanoid separation mentioned earlier, ionic liquid extraction and separation of Ce from other lanthanoids (particularly Pr) relies on the selective oxidation of Ce^3+^ to Ce^4+^ while other lanthanoids remain in their trivalent state. In one investigation (Gras et al. 2017), Ce was separated from lanthanoids of similar mass (La, Pr and Nd) using two different ionic liquids: trihexyltetradecylphosphonium bis(trifluoromethansulfonyl)imide ([P_66614_][NTf_2_]) and 1-methyl-1-butylpyrrolidinium bis(trifluoromethanesulfonyl)imide ([C_1_C_4_Pyrr][NTf_2_]). Initial studies indicated that [C_1_C_4_Pyrr][NTf_2_] exhibited better Ce^4+^ extraction efficiency by a factor of two, and thus was selected as the ionic liquid extractant for subsequent separations. A mixture of trivalent lanthanoid salts (La, Ce, Pr and Nd) was treated with oxygen under alkaline conditions (2 M NaOH, 30 °C, 3 h) to selectively oxidise Ce^3+^ to Ce^4+^ (87.1% Ce^3+^ oxidised to Ce^4+^) and cause the precipitation of Ce(OH)_4_, while the other unoxidized lanthanoids precipitated out as their trivalent hydroxide salts. The precipitated salts were filtered, washed and subsequently dissolved in HNO_3_ and the aqueous lanthanoid salt mixture formed was mixed with the ionic liquid ([C_1_C_4_Pyrr][NTf_2_]), stirred for 20 min and centrifuged at 6000 rpm for 10 min to facilitate the selective extraction of tetravalent Ce into the ionic liquid phase (Gras et al. 2017). The two phases were then separated and the Ce^4+^ stripped from the ionic liquid using dilute HNO_3_. The lanthanoids in their trivalent state are very poorly extracted into the ionic liquid due to their inability to form negatively charged complexes with nitrate ions that are soluble in the ionic liquid, whereas the tetravalent Ce is speculated to form a negatively charged polynitratoceriate(IV) complex which is readily extracted into the ionic liquid phase (Gras et al. 2017). Distribution coefficients (D) for Ce^4+^ and separation factors (β) for Ce^4+^ versus other Ln^3+^ ions were found to depend significantly upon the HNO_3_ concentration of the aqueous phase and the concentration of Ce^4+^ in the aqueous phase, with highest D and β values being obtained when higher HNO_3_ aqueous phase concentrations were used in combination with low Ce^4+^ concentrations (Gras et al. 2017). In an exemplar protocol for the separation of Ce from other lanthanoids using [C_1_C_4_Pyrr][NTf_2_], experimental parameters of 3.45 M HNO_3_ and 0.01 M Ce^4+^ were chosen to optimise the distribution coefficients and separation factors without significantly compromising the overall throughput of the method. Using these conditions, the protocol successfully achieved a Ce^4+^ distribution coefficient of 74.9 and Ce^4+^/Ln^3+^ separation factors on the order of 10^4^. Issues with the overall efficacy of this separation approach arose during the oxidation step, during which only 87.1% of total Ce^3+^ is oxidised to Ce^4+^ (Gras et al. 2017). This was attributed to the slow kinetics of the oxidation reaction caused by the low solubility of Ce_2_(SO_4_)_3_ in basic conditions and the imposed 3 h reaction time. Ultrasonication and longer oxidation times are expected to increase the overall oxidation percentage and thus total separation efficiency of the method (Gras et al. 2017). Despite this, 98.8% of the Ce^4+^ produced by the oxidation reaction was extracted and recovered after only one extraction and stripping cycle, while repeating the procedure resulted in 99.5% of the Ce^4+^ being recovered (Gras et al. 2017). Thus, with the requisite improvements to the oxidation reaction to maximise the oxidation percentage, near quantitative extraction and recovery of Ce from a mixture of similar-mass lanthanoids is highly feasible. However, while these separation results are promising for an ideal solution with similar Ln^3+^ concentrations, the application of this protocol to the separation of bulk target Ce from small amounts of ^143^Pr in a macro–micro component system for radionuclide production is yet to be realised, and has the potential to encounter extraction efficiency issues due to the observation that distribution coefficients for Ce^4+^ decrease with increasing Ce^4+^ concentration.

## ***Thulium: ***^***170***^***Tm***

In addition to ^153^Sm receiving considerable attention and subsequent approval for bone pain palliation treatment, ^170^Tm has been proposed as a suitable radionuclide for bone pain palliation due to favourable decay characteristics and an advantageous half-life (t_1/2_ = 128.6 d). Other radionuclides currently used for bone pain palliation such as ^89^Sr and ^153^Sm, while clinically beneficial, either still exhibit certain undesirable decay properties or lack certain desirable properties which have detracted from their overall suitability for bone pain palliation applications. The use of ^89^Sr in the form of [^89^Sr]SrCl_2_ has typically encountered issues with dose burden, due to the higher-energy *β*^−^-particles emitted by ^89^Sr causing bone marrow suppression (Vats et al. 2013). Furthermore, the absence of an imageable γ-emission has made dosimetry, biodistribution and pharmacokinetic studies more challenging since they cannot be conducted concurrently. In the case of ^153^Sm, while a lower *β*^−^-particle energy compared to ^89^Sr has alleviated issues relating to patient dose burden, logistical issues have arisen due to a shorter half-life which limits the distances the radionuclide can be transported before substantial decay and loss of overall activity has occurred.

In response to the identification of certain disadvantages for currently used bone pain palliation radionuclides, ^170^Tm has gained attention due to its longer physical half-life which increases the transportation and distribution radius and thereby the availability of the radionuclide (Goyal and Antonarakis 2012), while the lower *β*^−^-particle energies (β_max_ = 0.968 MeV) than the widely-used [^89^Sr]SrCl_2_ reduce the patient dose burden and consequently alleviate the potential for bone marrow suppression (Vats et al. 2013; Das et al. 2009). Moreover, ^170^Tm emits an imageable γ-photon (84 keV, 3.26%) which allows for dosimetry, biodistribution and pharmacokinetic data to be collected via SPECT imaging during treatment without any appreciable contribution to the patient dose burden (Das et al. 2009; Polyak et al. 2014). With advantages over both ^89^Sr and ^153^Sm, ^170^Tm has demonstrable potential as an alternative bone pain palliation agent while also displaying the requisite theranostic properties of simultaneous imaging and therapeutic capabilities (Vats et al. 2013; Das et al. 2009; Guerra Liberal et al. 2016; Shirvani-Arani et al. 2013). As a result, ^170^Tm is an interesting candidate for the application of theranostics to bone pain palliation.

Another advantage of ^170^Tm is the method by which it is produced, which provides a cost-effective production and purification pathway to access the target radiolanthanoid at sufficient activity yields using moderate flux nuclear reactors (Vats et al. 2013; Das et al. 2009; Danon et al. 1998; Neves et al. 2002). This is due to the fact that ^170^Tm is typically produced by means of the thermal neutron bombardment of ^169^Tm (100% natural abundance) via the ^169^Tm(n,γ)^170^Tm nuclear reaction, which exhibits a relatively high thermal neutron capture cross section (σ_th_ = 109 barns) as well as a high resonance integral (σ_RI_ = 1548 barn) (Das et al. 2009; Danon et al. 1998; Linden et al. 1974). An illustrative protocol for ^170^Tm production involved the use of powdered ^169^Tm_2_O_3_ targets and a thermal neutron flux of 6 × 10^13^ n/cm^2^/s for 60 days, culminating in a ^170^Tm specific activity of 6.36 GBq/mg. At EOB, the target was dissolved in 1 M HCl with heating to aid dissolution, after which the solution was evaporated to near dryness and reconstituted as the desired chloride salt in double-distilled water (Das et al. 2009). The final [^170^Tm]TmCl_3_ formulation exhibited a radiopurity of 100%, ascertained by the absence of any peaks in the γ-spectrum that did not correspond to ^170^Tm decay. In addition, the absence of radionuclidic impurities with substantially longer half-lives was confirmed by allowing the ^170^Tm to decay for 7 half-lives (~ 2.5 years) and monitoring the γ-spectrum which did not reveal any peaks that were not attributable to ^170^Tm (Das et al. 2009). This ^170^Tm formulation was used to successfully radiolabel EDTMP with > 99% radiochemical purity, and a normal Wistar rat model was used for dosimetric, biodistribution and scintigraphic imaging studies of the resultant [^170^Tm]Tm-EDTMP complex. Results showed significant preferential accumulation in the skeleton while other tissues and organs exhibited low to negligible uptake, positively highlighting the targeting ability of the complex (Das et al. 2009).

Notably, when employing neutron bombardment on ^169^Tm targets for the production of ^170^Tm, special attention must be paid to the irradiation parameters to minimise the formation of long-lived ^171^Tm impurities (t_1/2_ = 1.92 y) (Sahiralamkhan et al. 2016). The formation of unwanted ^171^Tm is the result of a double neutron capture event on the target ^169^Tm via the ^169^Tm(n,γ)^170^Tm(n,γ)^171^Tm nuclear reaction due to the similarly high cross section of the second neutron capture on ^170^Tm (σ = 92 barns c.f. σ = 109 barns). Consequently, when irradiated under medium to high neutron flux conditions, appreciable amounts of ^171^Tm are formed which will remain as contaminants long after the desired ^170^Tm has decayed due to its much longer half-life (Sahiralamkhan et al. 2016). This poses significant clinical translation issues for formulations of ^170^Tm due to the contaminant ^171^Tm exhibiting vastly different *β*^−^-decay and γ-emission properties, with a maximum *β*^−^-particle energy of only 97 keV (approximately 10% of ^170^Tm *β*^−^-particle energy) and a very low abundance γ-emission energy (66.7 keV, 0.16%) (Sahiralamkhan et al. 2016; Kajan et al. 2018; Tishchenko et al. 2015). Furthermore, due to its identical chemical nature, ^171^Tm cannot be separated from the desired ^170^Tm using traditional chemical means such as chromatography or extraction methodologies and consequently the specific activity and radiopurity of the final formulation are adversely affected. At higher neutron fluxes and longer irradiation periods, a greater degree of transmutation of ^170^Tm to ^171^Tm is observed, and as a result, ^171^Tm impurities are formed at significantly higher levels which impact upon the radiopurity and dilute the specific activity of the final formulation. Consequently, higher neutron fluxes on the order of 10^14^ and longer irradiation periods of > 120 days are not deemed ideal for preparation of clinically applicable ^170^Tm (Sahiralamkhan et al. 2016). Optimum irradiation conditions that achieve sufficient specific activity of ^170^Tm while essentially eliminating the by-production of ^171^Tm could be theoretically achieved using a neutron flux of 5 × 10^13^ n/cm^2^/s for a 15 day irradiation period, which would result in ~ 1.42 GBq/mg ^170^Tm with a radiopurity of > 99.9% (Sahiralamkhan et al. 2016). Such conditions were employed in the production of ^170^Tm for EDTMP radiolabelling, with ~ 1.52 GBq/mg ^170^Tm being produced at > 99.9% radiopurity suitable for bone pain metastatic applications. Subsequent radiolabelling of EDTMP was achieved at > 98% yield, equivalent to ~ 32.3 GBq/mM for [^170^Tm]Tm-EDTMP (Sahiralamkhan et al. 2016). This formulation was deemed suitable for a preliminary clinical study on a human male (73 y) with skeletal metastases arising from primary prostate carcinoma. 185 MBq of [^170^Tm]Tm-EDTMP was administered and the patient was imaged 24 h after administration whereby significant localised preferential uptake in metastatic skeletal lesions was observed, comparable to that of a [^99m^Tc]Tc-MDP bone scan (740 MBq, imaged 4 h after administration) (Sahiralamkhan et al. 2016). Compared to earlier production of ^170^Tm, which used a slightly higher neutron flux and a longer irradiation period (Das et al. 2009), ~ 4 times lower overall specific activity was obtained with the optimised parameters, however the final formulation was still deemed suitable for clinical applications in bone pain palliation (for which lower specific activity formulations are appropriate) and the shorter irradiation time has the potential to improve overall production and transportation logistics (Sahiralamkhan et al. 2016). Importantly however, the irradiation parameters employed by the earlier study afforded a [^170^Tm]TmCl_3_ product with radiopurity of practically 100%, hereby demonstrating that slightly higher neutron fluxes with longer irradiation times are capable of affording a product with higher specific activity than those obtained using the optimised parameters without necessarily compromising the final radiopurity (Das et al. 2009). The feasibility of this approach is also corroborated by the theoretical calculations reported in the more recent investigation (Sahiralamkhan et al. 2016).

**Fig. 12:**
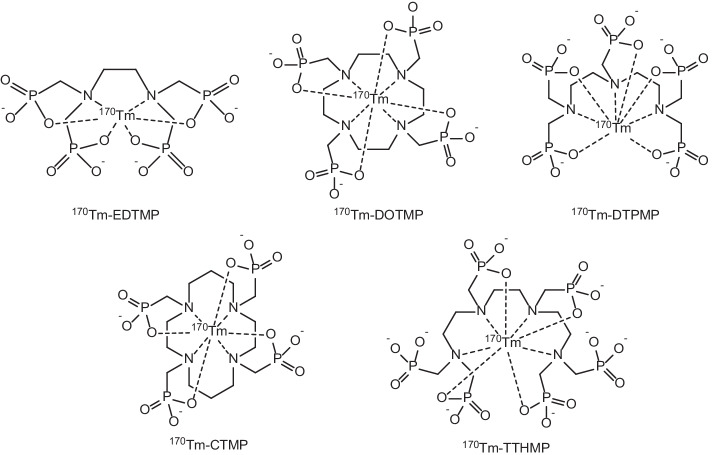
Structures of ^170^Tm-labelled phosphonate chelators

Further preclinical work towards the implementation of ^170^Tm in bone pain palliation applications has been undertaken by radiolabelling a series of both cyclic and acyclic polyaminophosphonic acid-based chelators, namely EDTMP, diethylenetriaminepentamethylenephosphonic acid (DTPMP), triethylenetetraminehexamethylenephosphonic acid (TTHMP), 1,4,7,10-tetraazacyclododecane-1,4,7,10-tetramethylenephosphonic acid (DOTMP) and 1,4,8,11-tetraazacyclotetradecane-1–4-8–11-tetramethylenephosphonic acid (CTMP) (see Fig. 12) (Vats et al. 2013; Shirvani-Arani et al. 2013; Shirmardi et al. 2020; Das et al. 2017). Dosimtery, biodistribution, pharmacokinetic, scinitigraphic and SPECT imaging studies of these radiolabelled compounds have given a preponderance of promising preclinical data indicating the significant potential for ^170^Tm as a theranostic probe for bone pain palliation arising from various cancers metastasising to the skeleton (Vats et al. 2013; Shirvani-Arani et al. 2013; Shirmardi et al. 2020; Das et al. 2017). In one study, ^170^Tm was prepared using a ^169^Tm_2_(NO_3_)_3_ target and a neutron flux of 3–4 × 10^13^ n/cm^2^/s for 5 days, resulting in a final formulation with adequate specific activity and radiopurity (> 99.99%) suitable for subsequent radiolabelling EDTMP with a yield of > 99% and high in vitro stability (> 95% after two months incubation at RT). Biodistribution studies and SPECT imaging of the resulting complex in Wistar rats showed high uptake in the skeleton (70% of administered dose), low levels of uptake for other major organs, fast clearance from the blood and renal excretion (Shirvani-Arani et al. 2013). Other polyaminophosphonic acid chelators have been evaluated in another study to compare potential alternatives to the more widely used EDTMP (Vats et al. 2013). A ^169^Tm_2_O_3_ target was irradiated with a 7 × 10^13^n/cm^2^/s neutron flux for 60 days to produce 6.44 TBq/g of [^170^Tm]TmCl_3_ with radiopurity of 99.62%. After radiochemical experiments to ascertain the optimum complexation conditions for each ligand, they were radiolabelled with ^170^Tm resulting in radiochemical purities of > 98%, except for CTMP for which approximately 97% complexation was achieved. High in vitro stability was also observed for all complexes. Biodistribution of each complex was investigated using a Wistar rat model, in which each complex showed negligible uptake in all vital organs and other tissues and displayed significant selective uptake in the bone matrix, which was confirmed by scinitigraphy. Fast renal clearance was also observed for each metal complex (Vats et al. 2013). Of the four complexes prepared, the best performing compound displaying the greatest potential for further investigation and development for clinical applications was [^170^Tm]Tm-DOTMP, owing to the lower amount of ligand required to achieve > 98% radiolabelling yield over a large pH range of 2—10, and higher thermodynamic stability of the complex (Vats et al. 2013).

More recently, however, [^170^Tm]Tm-EDTMP has attracted more considerable research attention. Further preclinical studies and preliminary clinical investigations have afforded results that are indicative of the significant future potential of [^170^Tm]Tm-EDTMP as a theranostic agent in bone pain palliation (Shirmardi et al. 2020; Das et al. 2017). One illustrative example is the efficacy evaluation of a freeze-dried EDTMP kit for the in-house preparation of [^170^Tm]Tm-EDTMP which has provided encouraging results (Das et al. 2017).

## Conclusions

As the frontiers of theranostics continue to be explored, it is highly probable that Rare Earth radionuclides will play an increased role in future. Further research into optimising production methods for radionuclides that are already in use, as well as in the development pipeline, will facilitate more streamlined translation to clinical applications, however with more nuclear reactors being retired in the next decade and research focus shifting towards linear accelerator and cyclotron production methods, Rare Earth radionuclides that can be reliably and cost-effectively produced using linear accelerators and/or cyclotrons will most likely see greater application in the clinic. In particular, those Rare Earth radionuclides that can be produced at commercial or biomedical cyclotrons already in operation (e.g. certain isotopes of Sc, Tb, La and Er) will be favoured due to the added logistical convenience of minimal transportation or on-site production. With interest in *α*-emitters increasing, ^149^Tb will likely play a larger role in theranostics in alpha settings due to its accompanying γ-emission for SPECT, however more facilities with ^149^Tb production capabilities, most notably high intensity ^3^He beamlines and mass separation capabilities for proton spallation, will be required in order to produce the desired quantities of ^149^Tb at reasonable cost for future clinical work. Likewise, theranostically matched Tb radionuclides for ^149^Tb will likely gain traction, however a greater emphasis on linear accelerator production methods for Tb isotopes will likely be required, as well as greater focus on mass separation methods to achieve the requisite radionuclidic purities when using proton spallation on Ta-foil for Tb isotope production. Rare Earth Auger emitters possess the interchangeability in chelator structures that would be advantageous for clinical evaluation of theranostic protocols, however for radionuclides such as ^165^Er further isotope production optimisation is necessary. As such, for the production of radionuclides such as ^153^Sm and ^165^Er where target material separation and/or impurity separation proves more challenging when using more conventional approaches, mass separation methods (both on-line and off-line) will likely play a more prominent role in future purification protocols for such radionuclides, due to promising indications that much higher radionuclidic purities are potentially attainable. It must, however, also be noted that the separation efficiencies of current mass separation methods require further optimisation in order to achieve these desired outcomes in the future. ^166^Ho has also been presented as a versatile radionuclide in many cancer settings, and as Rare Earth bifunctional chelators are explored further, the remit of this radionuclide will likely be expanded further. In addition, as radionuclide production methods and purification improve the overall activity yield and purity of desired Rare Earth radionuclides, the versatility of interchangeability in established ligand structures (e.g. DOTA, EDTMP and related structures) will expand the library of available radionuclides for therapy (*α*, *β*, γ) and imaging (MRI, PET, SPECT) to include more Rare Earth radiosiotopes, which will allow for a more personalised theranostic approach to cancer treatment in years to come. While the outlook for future innovation in the field of Rare Earth radionuclides for theranostic applications is positive, the retirement of aging reactors around the world necessitates a shift in research focus to linear accelerator and cyclotron production methods to maintain pace with the growing demand for radionuclides for nuclear medicine applications. Consequently, an increase in linear accelerator and cyclotron infrastructure will be required to meet future production demands.

## Data Availability

Not applicable.

## Abbreviations

AE: Auger electron
CERN-MEDICS: Medical isotopes collected from ISOLDE
CTMP: 1,4,8,11-Tetraazacyclotetradecane-1-4-8-11-tetramethylenephosphonic acid
DCH18C6: Dicyclohexano-18-crown-6
DO3Apic: 10-((6-Carboxypyridin-2-yl)methyl)-1,4,7,10-tetra-azacyclododecane-1,4,7-triacetic acid
DOTA: 1,4,7,10-Tetraazacyclododecane-1,4,7,10-tetraacetic acid
DOTA-OSSu: *N*-Hydroxysulfosuccinimidyl DOTA
DOTA-NOC: DOTA-1-NaI^3^-octreotide
DOTA-TATE: DOTA-octreotate
DOTA-TOC: DOTA-octreotide
DOTMP: 1,4,7,10-Tetraazacyclododecane-1,4,7,10-tetramethylenephosphonic acid
DTPMP: Diethylenetriaminepentamethylenephosphonic acid
DUPA: 2-[3-(1,3-Dicarboxypropyl)ureido]pentanedioic acid
EDTMP: Ethylenediaminetetra(methylene phosphonate)
EOB: End of bombardment
EOI: End of irradiation
Gd-BT-DO3A: Gadolinium(III)-tris(carboxymethyl)-1,4,7,10-tetraazacyclododecane-butriol
Gd-DTPA: Gadolinium(III)-diethylenetriaminepentaacetic acid
HA: Hydroxyapatite
HDEHP: Di-(2-ethylhexyl)phosphoric acid
HEH[EHP]: 2-Ethylhexylphosphonic acid mono-2-ethylhexyl ester
α-HIBA: α-Hydroxyisobutyric acid
HPGe: High purity germanium
ICP-OES: Inductively coupled plasma optical emission spectrometry
ISAC: Isotope Separator and Accelerator
ISOLDE: Isotope seperator on line device
LET: Linear energy transfer
macropa: *N*,*N*’-Bis[(6-carboxy-2-pyridil)methyl]-4,13-diaza-18-crown-6
MDP: Methylenediphosphonate
MELISSA: MEDICIS’ Laser Ion Source Assembly
MeO-DOTA: Methoxy-DOTA
MRI: Magnetic resonance imaging
PET: Positron emission tomography
PMMA: Polymethylmethacrylate
PSMA: Prostate-specific membrane antigen
PSMA-617: Vipivotide tetraxetan
RIT: Radioimmunotherapy
*p*-SCN-Bn-DOTA: S-2-(4-isothiocyanatobenzyl)-1,4,7,10-tetraazacyclododecanetetraacetic acid
SPECT: Single photon emission computed tomography
TBP: Tri-butylphosphate
TENDL: TALYS-based evaluated nuclear data library
TND: Tumour-to-normal-tissue distribution ratio
TRIUMF: Tri-University Meson Facility
TTHMP: Triethylenetetraaminehexamethylenephosphonic acid
